# Feature selection and association rule learning identify risk factors of malnutrition among Ethiopian schoolchildren

**DOI:** 10.3389/fepid.2023.1150619

**Published:** 2023-07-06

**Authors:** William A. Russel, Jim Perry, Claire Bonzani, Amanda Dontino, Zeleke Mekonnen, Ahmet Ay, Bineyam Taye

**Affiliations:** ^1^ Department of Biology, Colgate University, Hamilton, NY, United States; ^2^ Department of Computer Science, Colgate University, Hamilton, NY, United States; ^3^ Department of Mathematics, Colgate University, Hamilton, NY, United States; ^4^ Institute of Health, School of Medical Laboratory Sciences, Jimma University, Jimma, Ethiopia

**Keywords:** machine learning, association rule learning, malnutrition, risk factor analysis, school-aged children

## Abstract

**Introduction:**

Previous studies have sought to identify risk factors for malnutrition in populations of schoolchildren, depending on traditional logistic regression methods. However, holistic machine learning (ML) approaches are emerging that may provide a more comprehensive analysis of risk factors.

**Methods:**

This study employed feature selection and association rule learning ML methods in conjunction with logistic regression on epidemiological survey data from 1,036 Ethiopian school children. Our first analysis used the entire dataset and then we reran this analysis on age, residence, and sex population subsets.

**Results:**

Both logistic regression and ML methods identified older childhood age as a significant risk factor, while females and vaccinated individuals showed reduced odds of stunting. Our machine learning analyses provided additional insights into the data, as feature selection identified that age, school latrine cleanliness, large family size, and nail trimming habits were significant risk factors for stunting, underweight, and thinness. Association rule learning revealed an association between co-occurring hygiene and socio-economical variables with malnutrition that was otherwise missed using traditional statistical methods.

**Discussion:**

Our analysis supports the benefit of integrating feature selection methods, association rules learning techniques, and logistic regression to identify comprehensive risk factors associated with malnutrition in young children.

## Introduction

Current epidemiological efforts targeted toward low- and middle-income countries focus on the double burden of malnutrition, which is defined as the simultaneous occurrence of underweight and overweight outcomes in a population (1). While undernutrition and overnutrition have historically occupied separate populations, their co-occurrence has become more frequent within lower-income populations as economic development and urbanization occur (2–5). Despite increases in overweight and obesity (5), undernutrition remains a significant concern in developing countries, affecting more than 150 million children (6). This emphasizes the need to better understand factors shaping undernutrition in these developing contexts so that more effective interventions can be established. The WHO uses several metrics to assess malnutrition, describing low height-for-age as stunting, low body-mass index (BMI)-for-age as thinness (7), and low weight-for-age as underweight. Stunting reveals chronic nutritional deficiencies (8), while thinness is an acute form of malnutrition, and underweight is a composite metric to assess both acute and chronic malnutrition. Nutritional deficiencies in early and intermediate childhood have developmental consequences on physical development (9), cognitive performance (10, 11), and lifespan (12), resulting in a greater risk of morbidity, mortality, and reproductive hindrance (12–14).

Africa accounts for over one-third of global stunting cases and is one of the few regions where stunting prevalence is not significantly decreasing (6, 15). In Africa, despite a reduction in malnutrition-related disability-adjusted life years (DALYs), this continent still comprises the largest share of malnutrition DALYs and has the highest age-standardized death rate from malnutrition (16). Child undernutrition is most highly concentrated in the eastern and southern regions of Africa (17). Ethiopia, located in the eastern part of Africa, ranks among other countries as having one of the highest prevalences of undernutrition, as an estimated 40% of children are stunted and 25% of children are underweight (17). Ethiopia is still struggling to meet the United Nations (UN) Sustainable Development Goal 2 (“Zero Hunger”) by 2030 (18), highlighting the need to implement more effective interventions. Therefore, it is crucial to analyze risk factors for undernutrition in this region.

Several previous studies have demonstrated how complex the etiology of malnutrition is in Ethiopia (19–23). Two systematic reviews and metanalyses of epidemiological studies showed a combination of environmental, demographic, and socioeconomic statuses as significant determinants of malnutrition (24, 25). For example, access to nutritious food and sanitation and hygiene factors have been identified as important environmental variables, residential area, child age and sex as important demographic factors, and maternal education status and socioeconomic status as important economic factors (24, 25). Despite these insights, there is still a great need for research to further analyze these trends and clarify the mechanisms by which they may be associated with undernutrition (24, 25).

Additionally, these studies have all relied solely on logistic regression methods to examine the determinants of under-nutrition (24, 25). However, traditional logistic regression models alone may struggle to produce accurate findings since large datasets with many variables are prone to overfitting (26). This could be improved by integrating machine learning techniques that utilize advanced mathematical and statistical methods to discern patterns within a dataset (27). These approaches appear to be a promising alternative to logistic regression since they avoid overfitting (28, 29). ML techniques have previously been used to identify risk factors for parasitic infections, congestive heart failure, diabetes, overweight/obesity, and dementia (30–34). Moreover, several recent studies have used ML approaches to effectively predict undernutrition outcomes in Bangladesh, India, and Ethiopia (35–39). These studies indicate that ML methods such as feature selection may be able to identify risk factors effectively (36). Despite these advances, most studies failed to implement association rule learning, another promising ML method that may facilitate the identification of risk factors. Association rule learning has previously shown promise in predicting disease co-occurrences and risk factors for parasitic infection (34, 40). Altogether, these ML methods could identify important risk factors for undernutrition, and potentially provide crucial insights into how the co-occurrence of variables may lead to undernutrition.

To the best of our knowledge, no study has used multiple ML techniques in conjunction with logistic regression to investigate risk factors for undernutrition outcomes. Such analysis could improve targeted public health interventions by uncovering novel variables associated with stunting, underweight, or thinness in Ethiopian schoolchildren. In this study, we used ML feature selection and association rule learning with logistic regression to identify risk factors for stunting, underweight, and thinness in Ethiopian school-aged children.

## Materials and methods

### Data collection

**Figure 1:**
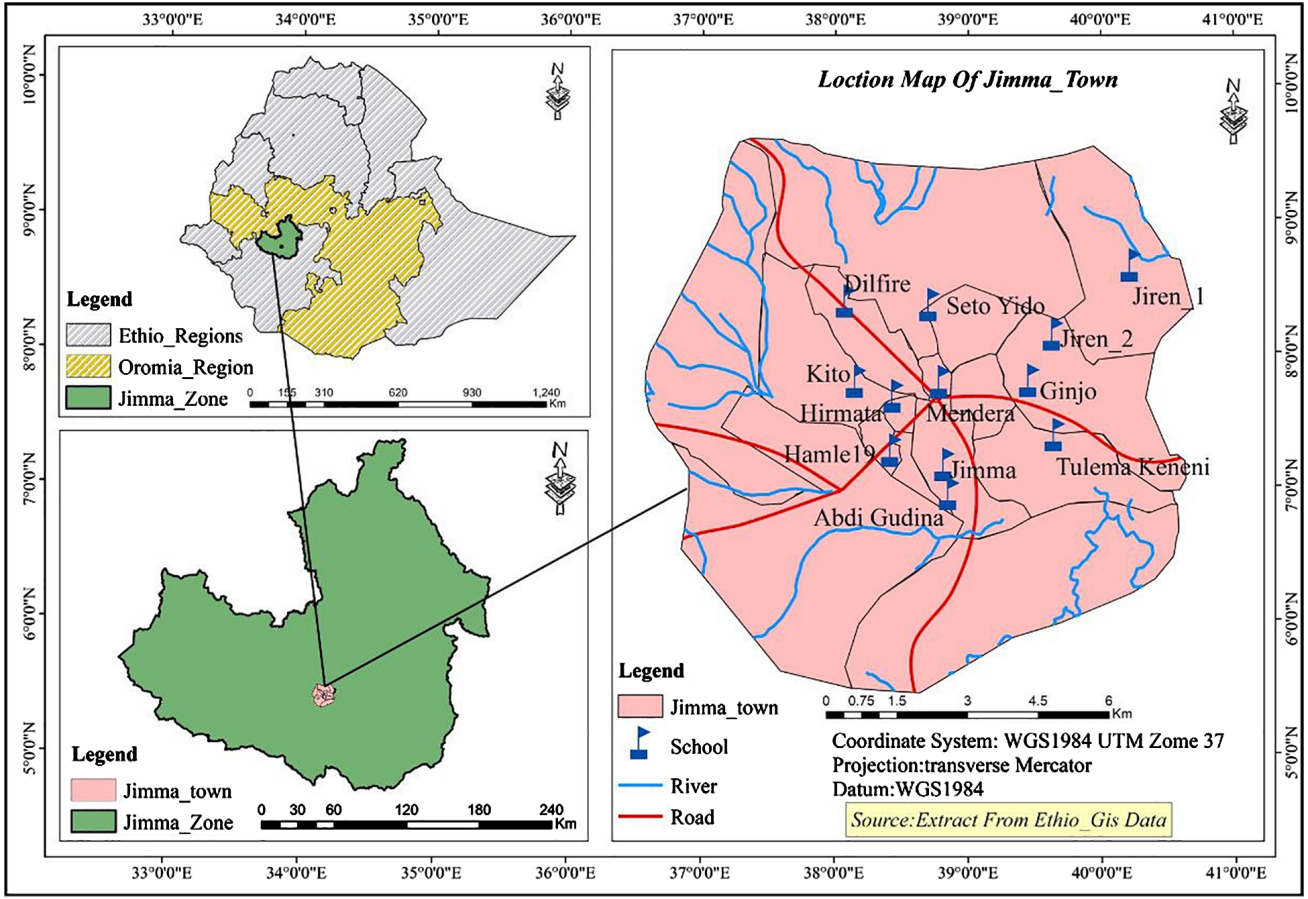
Map represents study location and primary schools surveyed in this study. Ten primary schools were selected based on their geographical distribution in the town to represent all population. These schools included Jiren_1, Jiren_2, Ginjo, Tulema Keneni, Abdi Gudina, Jimma, Mendera, Seto Yido, Dilfire, Kito, Hirmata and Hamle 19 primary schools.

This project was carried out in Jimma Town, Ethiopia. Jimma is located 352 km southwest of Addis Ababa with an altitude of 2,450 m above sea level and average temperatures ranging from 15°C to 18°C (Figure 1). In this area, there is a low level of sanitation practiced and many households lack access to clean drinking water. The study catchment area included 14 public elementary schools, all of which were surveyed, resulting in a total of 1,036 participants (498 males, 538 females) (Figure 1). Students were randomly selected and had parental consent to participate after a complete explanation of the experiment and objective. Each parent was given adequate time to think about and discuss their decision as to whether they wanted their child to participate. Data was collected in a 2021 survey titled “Understanding gut-microbiome interactions following mass deworming against soil-transmitted helminths (STHs) among young Ethiopian schoolchildren”. The survey included a series of yes/no questions relating to sociodemographic and behavioral factors in addition to pertinent medical history including but not limited to sickness, receiving a deworming drug, and BCG vaccination status. The survey also included anthropometric measures used to calculate malnutrition outcomes.

### The list of risk factors

From the administered survey, we obtained data on the study populations' demographic, socioeconomic, biological, and behavioral characteristics. The complete list of risk factors used in this study is provided in Supplementary Table S2.

### Outcome definition

Stunting, underweight, and thinness were determined using the World Health Organization's Growth Reference Study Group guidelines by AnthroPlus package in R (41). This package uses an individual's sex, age, height, and weight and computes a height-for-age (41), weight-for-age (42), and BMI-for-age metric (BAZ). It then compares this metric to the WHO Reference 2007 for 5–19 years and returns a z-score indicating the deviation of this individual from the mean of the reference population. These z-scores are continuous variables and are used in the multivariate linear regression described below. To determine the stunting, underweight, and thinness classes for feature selection, the z-scores were converted into a binary measure, where “0” indicates a z-score greater than −2 and “1” indicates a z-score less than −2 (an individual with stunting, underweight, or thinness).

### Data imputation

Survey data often have incomplete responses, dependent on a participant's willingness or ability to answer certain questions. Out of 41,280 total values, 674 values were missing for stunting and underweight, and 427 values were missing for thinness. Even though there were relatively few missing values, minimizing missing values as much as possible is necessary to produce the most effective ML model, as missing values can introduce selection bias (43). To ensure that the model is unbiased, the data were initially preprocessed using K-nearest neighbors (K-NN) imputation with five nearest neighbors, which is a standard number of neighbors used for data imputation (44). K-NN imputation was chosen instead of other single imputation techniques, like mean or median substitution, because of its ability to determine missing data points, based on similar samples in the dataset (44). Since the dataset contains multiple types of data (categorical and numerical), the K-NN imputation was conducted once to impute numerical variables and once to impute categorical variables. Imputation has been only applied to samples with less than 5% missing values. Four samples were either too young or too old to calculate undernutrition metrics, thus 1,032 out of 1,036 entries could be used in this study. To check the performance of K-NN imputation, we also performed random forest imputation and compared the overlap in values between the two methods. There was a 90.4% overlap in values for stunting, a 90.1% overlap for underweight, and an 88.4% overlap for imputed thinness data (Supplementary Table S1), showing that K-NN imputation was consistent with other imputation methods.

### One hot encoding

The data consists of a mix of categorical, numerical, and binary data. The categorical variables were encoded using one-hot encoding, which takes the potential risk factors with more than two groups and creates multiple factors from their class distribution. One-hot encoding is necessary before performing logistic regression since regression is a distance-based method and categorical variables must be encoded to be analyzed by distance-based methods (45). After one-hot encoding, the reference category was removed to account for multicollinearity and redundancy in the dataset.

### Logistic regression

Univariate and multivariate logistic regression was performed for stunting, underweight, and thinness outcomes. The *p*-values for risk factors were adjusted for multiple testing using the Benjamini-Hochberg procedure (46). Risk factors identified were compared using this statistical method to significant variables identified by three feature selection methods. Multivariate linear regression was also performed in this comparative analysis to identify other potentially important variables.

### Feature selection

Feature selection algorithms were used to identify key determinants of different malnutrition outcomes. Chi-square and Monte-Carlo were used as ranking-based feature selection techniques alongside minimum redundancy maximum relevance (mRMR) and joint mutual information (JMI) subset-based feature selection methods. The chi-square method calculates the association between risk factors and undernutrition outcomes using the chi-squared score (47). Monte-Carlo is a commonly used feature selection method (48–50), which ranks features based on their contribution to the undernutrition outcome based on a relative importance metric (51). Subset-based methods choose a subset of a dataset's original features that collectively possess a good predictive ability (52). MRMR and JMI both select features that have the strongest relationships with an outcome and the weakest relationship with other risk factors by evaluating and comparing these two different interactions. The top 10 features with the highest importance level from the ranking- and subset-based feature selection methods and multivariate linear regression were used in further analysis.

Based on a literature search, feature selection was also performed for subgroups of the dataset that were suspected to have a large influence on the lifestyle of individuals. This subset analysis was performed across residence (urban vs. suburban/rural), age (up to 10 vs. older than 10), and sex (male vs. female) subsets to explore how population subsets are exposed to different undernutrition risk factors.

### Association rule learning

Association rule learning was used to discern risk factor combinations strongly associated with stunting, underweight, or thinness (53). Association rule learning gives support, confidence, and lift values for variable combinations. Support assigns a value based on the frequency that a rule occurs in the dataset and confidence indicates the amount of time that the rule is true. Lift approximates the association rule's strength, which is defined as the ratio of observed support to expected support in the instance that the risk factor and outcome are unrelated. *P*-values were also calculated for each rule to gauge the effect and significance of a rule's association with stunting, underweight, or thinness outcomes using Fisher's Exact test (54). We selected the rules that had the greatest significance values. This analysis was performed on the entire dataset, as well as age, residence, and sex population subsets. We also used the CART method to supplement rules provided by association rule learning (55). The CART method was used to obtain a decision tree for undernutrition outcomes, and this decision tree was transcribed into the format of our association rules for interpretability.

### Code and data availability

R programming language was used to write the study's code since it is user-friendly and contains advanced statistical learning libraries. Our source code is available on GitHub at https://github.com/CJPIV/SR2022Malnutrition. The principal investigator can provide access to the survey data upon reasonable request.

## Results

### Risk factors identified in logistic regression

To investigate stunting, underweight, and thinness outcomes, we performed univariate and multivariate logistic regression analyses (Supplementary Tables S2–4). Multivariate logistic regression revealed slightly increased odds of stunting in school-aged children as they grow older (OR = 1.1278, 95% CI = 1.0565–1.2045, adjusted *P* = 0.013) (Table 1). Females had about fifty percent lower odds of being stunted compared to males (OR = 0.5783, 95% CI = 0.4155–0.8016, adjusted *P* = 0.022). Similarly, having received childhood vaccinations reduced the odds of stunting (OR = 0.4452, 95% CI = 0.2634–0.7376, adjusted *P* = 0.028) (Table 1). Older children had nearly twice the odds of being underweight (OR = 1.8793, 95% CI = 1.3907–2.6027, adjusted *P* = 0.003) (Table 1).

**Table 1:** Multivariate logistic regression identifies significant risk and protective factors for undernutrition in Ethiopian school-aged children.

	Odds Ratio	95% CI	*P*-value (adj. P)
Stunting^a^			
Age	1.128	1.06–1.20	0.0003 (0.013)
Sex (F)	0.578	0.42–0.80	0.0011 (0.022)
Vaccination	0.445	0.26–0.74	0.0021 (0.028)
Underweight^b^			
Age	1.879	1.39–2.60	7.36E-05 (0.003)
Defecate in open field	0.359	0.12–0.90	0.0406 (0.833)
Thinness^c^			
Own sheep or goat	5.563	1.37–19.65	0.0102 (0.419)

Multivariate logistic regression was performed for each value for stunting, underweight, and thinness outcomes and then the *p*-value was corrected using the Benjamini-Hochberg correction.

^a^ Stunting was defined as HAZ <−2.

^b^ Underweight was defined as WAZ <−2.

^c^ Thinness was defined as BMIZ <−2.

### Machine learning risk factor analysis

ML feature selection complemented logistic regression, while also providing a novel approach to risk factor identification. For each feature selection method, variables were considered important if they appeared in the top 10 features for 90% of performed feature selection runs. Features that appeared in three or more feature selection methods across stunting, underweight, and/or thinness outcomes were considered important (Table 2). In line with regression findings, age was selected for stunting and underweight by mRMR, JMI, Monte-Carlo, univariate logistic regression, multivariate logistic regression, and multivariate linear regression (Table 2). Sex and vaccination status were selected for stunting by mRMR, JMI, multivariate logistic regression, and multivariate linear regression (Table 2). School latrine cleanliness, family size, and nail trimming habits were novel variables identified solely by feature selection chi-square or JMI methods (Table 2). However, the directionality of these associations is unclear, as these variables were not significant in the multivariate logistic regression.

**Table 2:** Feature selection identifies having a school latrine cleanliness, a larger family size, and nail trimming habits as potential factors associated with undernutrition.

	Stunting^a^							Underweight^b^							Thinness^c^						
	C	M	J	MC	U	M	L	C	M	J	MC	U	M	L	C	M	J	MC	U	M	L
Demographic factors																					
Age		√	√	√	√	↑	↑	√	√	√	√	√	↑	↑			√				↑
Sex	√	√	√		√	↓	↓														
Mother attended primary school														↓							
School latrine cleanliness	√							√							√						
Owns sheep or goat																				↓*	↑
Owns household pet		√					↑														
Family size			√							√							√				
Lives in urban environment																					↓
Medical factors																					
Vaccination status		√	√			↓	↓														
Deworming status																					↑
Takes any medication																√					
Has asthma		√														√					
Behavioral factors																					
Nail trimming	√							√							√						
Cooking with electricity							↓														
Open defecation										√			↓*								
Raw vegetable wash frequency										√											
Handwashing with soap														↑							
Handwashing with water only														↑							↑

Feature selection was performed with chi-square (C), MRMR (M), JMI (J), Monte-Carlo (MC), univariate logistic regression (U), multivariate logistic regression (M), and multivariate linear regression (L) for all variables. Only features identified more than three times across stunting, underweight, and thinness outcomes are considered important.

* Significance lost after Benjamini Hochberg *p*-value correction.

^a^ Stunting was defined as HAZ <−2.

^b^ Underweight was defined as WAZ <−2.

^c^ Thinness was defined as BMIZ <−2.

### Association rule learning

We used association rule learning to examine how variable co-occurrences may be associated with increased odds of stunting, underweight, and thinness. We observed unique trends in rules for stunting, underweight, and thinness (Table 3). Owning an animal frequently appeared alongside hygiene-related variables for stunting rules. For underweight, antibiotic use and open defecation were frequently found in rules that also contained related hygiene variables (i.e., walking barefoot, washing raw vegetables infrequently, cleaning oneself or clothes in a river). Similarly, open defecation was found with hygiene-related variables including handwashing with water only and nail trimming habits in multiple rules for thinness. Association rule learning using the CART method also revealed the co-occurrence of hygiene variables, in addition to variables identified by regression and feature selection (Supplementary Table S5).

**Table 3:** Association rule learning identifies co-occurring variables associated with stunting, underweight, and thinness.

^ ^	Support	Confidence	Lift	OR	*p*-value
Stunting ^a^					
Own household pet, don't use toilet paper, frequency of raw vegetable washing	0.018	0.39	1.72	2.29	0.006
Own chicken, don't use toilet paper, eat soil	0.012	0.39	1.72	2.24	0.029
Own chicken, don't use toilet paper, frequency of raw vegetable washing	0.012	0.39	1.72	2.24	0.029
Own chicken, latrine is outside, don't use toilet paper	0.012	0.39	1.72	2.24	0.029
Larger family size, own chicken, don't use toilet paper	0.012	0.39	1.72	2.24	0.029
Underweight ^b^					
Defecate in field, walk barefoot, antibiotics use	0.0052	0.33	3.64	5.20	0.041
Own housepet, antibiotics use, clean self or clothes in river	0.0069	0.25	2.73	3.50	0.050
Defecate in field, antibiotics use, wash hands with soap and water after latrine	0.0052	0.30	3.28	4.45	0.055
Defecate in field, antibiotics use, clean self or clothes in river	0.0052	0.27	2.98	3.89	0.071
Defecate in field, frequency of raw vegetable washing, antibiotics use	0.0052	0.25	2.73	3.45	0.088
Thinness ^c^					
Cook with electricity, defecate in field, nail trimming habits	0.0039	0.15	4.29	5.36	0.012
Defecate in field, walk barefoot, take antibiotics	0.0019	0.17	4.65	5.63	0.066
Don't use school latrine, wash hands with water after latrine, nail trimming habits	0.0019	0.17	4.65	5.63	0.066
Defecate in field, antibiotics use, wash hands with soap and water after latrine	0.0019	0.15	4.29	5.11	0.076
Own household pet, don't use school latrine, wash hands with water after latrine	0.0019	0.15	4.29	5.11	0.076

Association rules were computed using three or fewer variables on the left-hand side and rules with the highest lift values were selected for. Odds ratios and *p*-values were also calculated for association rules.

^a^ Stunting was defined as HAZ <−2.

^b^ Underweight was defined as WAZ <−2.

^c^ Thinness was defined as BMIZ <−2.

### Subset-adjusted findings

To analyze how risk factors may be constrained by larger determinants of health, we performed logistic regression and feature selection analyses for sex, residence, and age subsets. Using multivariate logistic regression, we observed the uneven distribution of risk factors in the population (Table 4). Older childhood age (10–18 years old) was again a risk factor, while having received vaccinations was a protective factor for the urban population subset. Interestingly, owning a household pet was a risk factor for females, urban, and children under 10 years old subsets; although findings remained significant only for the female subset following *p*-value correction. For underweight, older age was a significant risk factor exclusive to urban and male populations. Owning a household pet, having a house floor made of dust, open defecation, and older age also displayed subset-specific significance for underweight, although this significance was lost to *p*-value correction. Similarly, subset-specific findings for thinness which identified older age and owning a sheep (or goat) as risk factors specific to males did not remain significant after adjustment.

**Table 4:** Multivariate logistic regression for residence and age population subsets reveals significant risk and protective factors.

^ ^	Subset	AOR	95% CI	*P* (adj. P)
Stunting^a^				
Age	Urban	1.16	1.08–1.25	9.05E-5 (0.0036)
	Male	1.17	1.06–1.30	0.0023 (0.093)
	Female	1.13	1.02–1.24	0.014 (0.29)
Household pet	Urban	1.57	1.10–2.26	0.014 (0.14)
	Female	2.32	1.43–3.78	0.00069 (0.028)
	Up to 10	1.75	1.11–2.76	0.016 (0.22)
Received vaccination	Urban	0.42	0.24–0.72	0.0021 (0.027)
Sex (F)	Up to 10	0.55	0.34–0.88	0.013 (0.22)
Underweight^b^				
Age	Urban	1.82	1.30–2.63	0.00073 (0.029)
	Male	2.79	1.70–4.98	0.00017 (0.0067)
Household pet	Female	3.78	1.24–12.39	0.022 (0.80)
House floor made of dust	Female	0.22	0.044–0.89	0.043 (0.80)
Defecates in open field	Male	0.14	0.026–0.52	0.0080 (0.16)
Thinness^c^				
Age	Male	1.51	1.11–2.11	0.011 (0.15)
Own sheep or goat	Male	4.15	4.20–57.02	0.0023 (0.092)

Multivariate logistic regression was performed on urban vs. suburban/rural and up to 10 vs. 10 and older population subsets and variables that were significant prior to *p*-value adjustment were displayed.

^a^ Stunting was defined as HAZ <−2.

^b^ Underweight was defined as WAZ <−2.

^c^ Thinness was defined as BMIZ <−2.

**Figure 2:**
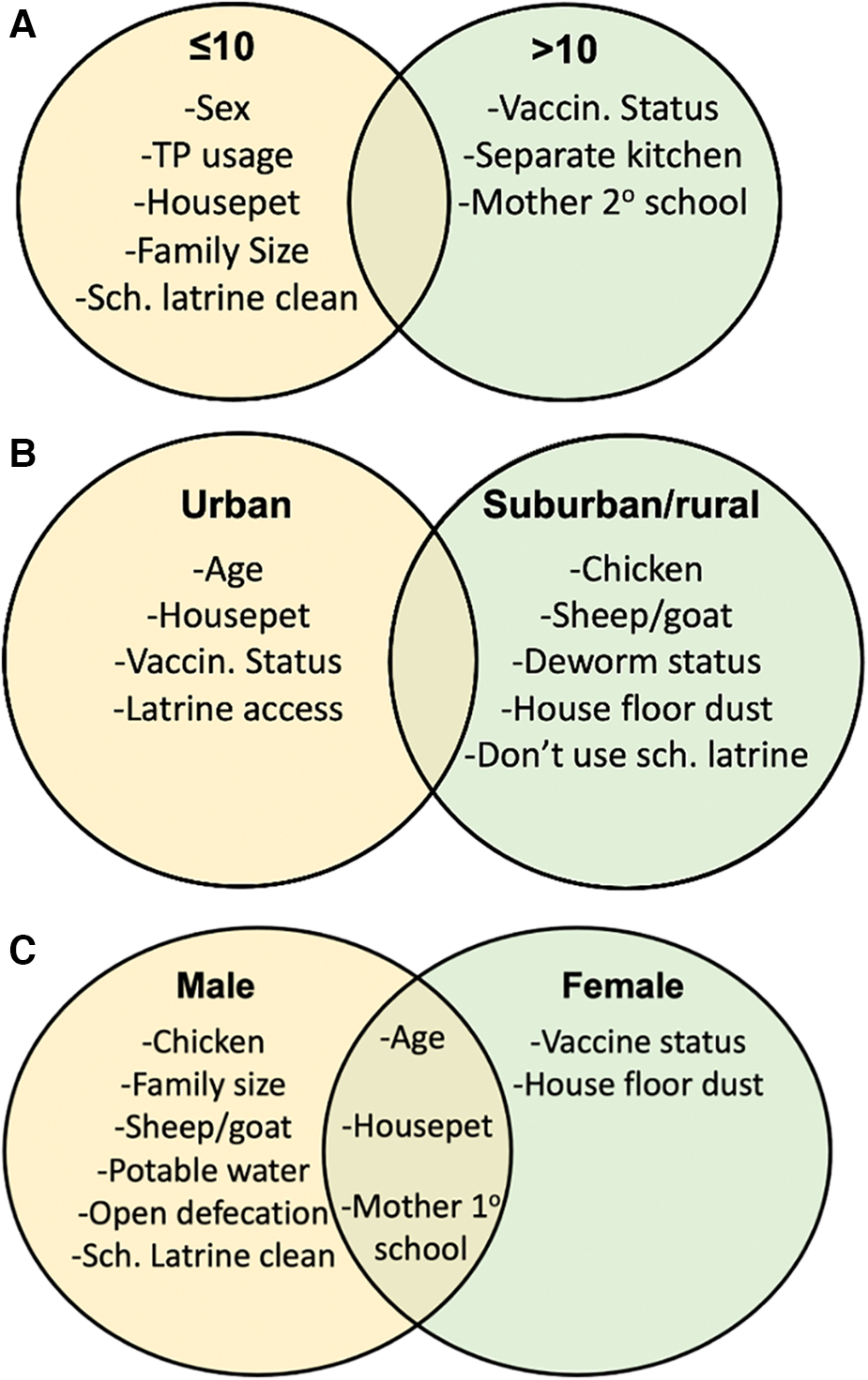
Feature selection for age and residence subsets identifies noticeable differences in important determinants of undernutrition. Feature selection was performed for age, residence, and sex subsets. Feature selection was performed for (A) ≤10 vs. >10, (B) urban vs. suburban/rural, and (C) male vs. female population subsets and summary tables were created to display variables important to each population subset.

Feature selection was also performed for sex, residence, and age subsets to examine how variables predictive of stunting, underweight, or thinness vary by these subsets. Again, variables were considered important for a feature selector if they appeared in the top 10 features for 95% of performed feature selection runs. In addition to identifying the same subset-specific variables observed in logistic regression, feature selection displayed interesting variable trends for different population subsets (Figure 2). Again, household pet ownership and sex were variables important to the up to 10 years old subset (Figure 2A). In addition, family size, toilet paper usage, school latrine cleanliness, and sex were important factors exclusive to children up to 10. Vaccination status, kitchen placement, and a maternal education status were important factors exclusive to children older than 10 (Figure 2A). For the urban subset, age, household pet ownership, and vaccination status were again present, while having a school latrine cleanliness was also identified (Figure 2B). Farm animals, a house floor made of dust, and deworming status were factors specific to suburban/rural residence (Figure 2B). Household access to potable water, large family size, open defecation, owning a farm animal, and school latrine cleanliness were important factors for males, while vaccination status and having a house floor made of dust were important factors for females (Figure 2C). Age, owning a household pet, and maternal education status were important for both male and female subsets (Figure 2C). Subset association rule learning was also performed for age, residence, and sex subsets, and variables identified by these previous methods also tended to appear in association rules (Supplementary Table S6). Like the association rule learning analysis that was applied on the entire dataset, hygiene variables and behaviors such as spending time in rivers, antibiotic use and owning animals appeared in several rules (Supplementary Table S6).

## Discussion

In our study, we performed ML approaches in conjunction with logistic regression to explore potential risk factors associated with malnutrition. Both multivariate logistic regression and feature selection identified older age, sex, and vaccination status as important risk factors for stunting, underweight, and/or thinness. In addition, feature selection identified novel factors for these detrimental outcomes, such as a larger family, nail trimming habits, and school latrine cleanliness. Association rule learning displayed interesting hygiene and animal variable co-occurrences. Our subset analysis revealed noticeable differences in significant factors for different population subsets. Collectively, these findings offer new insights into the complexity of stunting, underweight, and thinness in Ethiopian schoolchildren, suggesting that future studies should seek to use these complementary ML methods to provide a more comprehensive analysis.

Our finding that older age is associated with greater odds of stunting and underweight is consistent with previous literature (56–59), and is likely the result of the increased nutritional demands of children as they transition into adolescence (60). Males tend to require greater nutritional intake to maintain muscle mass, increasing their sensitivity to environmental conditions (61). Biological differences may also be exacerbated by cultural norms in which males often engage in strenuous physical labor compared to females who perform household-related tasks and are often in closer proximity to food (56). Additionally, vaccination appeared to have a significant protective role against stunting, which has also appeared in previous studies (62, 63) and suggests that children face long-term health consequences based on their susceptibility to infection if unvaccinated. Nandi et al. (2019), found that Ethiopian, Indian, and Vietnamese school-aged children who received early-life measles vaccination (6–18 mo.) had greater anthropomorphic measurements than matched unvaccinated children, suggesting the long-term benefits of vaccination (64).

Feature selection complemented logistic regression while identifying novel factors predictive for stunting, underweight, and thinness, revealing its value when used alongside traditional statistical methods. Large family size, school latrine cleanliness, and nail trimming habits were identified as novel predictors of stunting, underweight, and thinness. Bazie et al. (2021) suggested that increases in family size contribute to spreading resources more thinly among a greater number of children and predisposing them to smaller and less diverse diets (65). Reinforcing this conclusion, high physiological density, or number of persons per unit of agricultural land, has previously been associated with a greater likelihood of undernutrition (66).

School latrine cleanliness and nail trimming habits are likely representative of sanitation and hygiene access. Previously, having access to a clean latrine has been identified as an important determinant of child malnutrition (67, 68). Repeated exposure to fecal matter in an unclean school latrine may result in undernutrition through enteric dysfunction, as pathogenic bacteria present in the feces, such as *E. Coli*, may damage the intestinal mucosa and prevent nutrient absorption (69, 70). Additionally, parasites such as *Ascaris lumbricoides* shed eggs in feces. Exposure to these eggs can lead to subsequent parasitic infection, which is strongly associated with malnutrition (71, 72). Long nails can collect soil or fecal material, inadvertently leading to the collection of parasites. In fact, previous research in Jimma, Ethiopia has found that dirt trapped in nails contributed to helminth infection (73, 74), and that odds of parasitic infection increased as nail trimming decreased (75). Once on the nails, a parasitic infection may occur through the fecal-oral route as children bring their hands close to their mouths. As parasitic infection occurs, children may have a decreased appetite (76), greater nutrient loss due to vomiting (77), or exhibit malabsorption of nutrients (78).

Association rule learning was performed to identify variable co-occurrences associated with a greater likelihood of stunting, underweight, and thinness. For stunting, owning a chicken or house pet was frequently found with hygienic variables including toilet paper usage status and handwashing habits before eating. Chickens and house pets may serve as reservoirs for enteric bacteria such as *E. coli, Campylobacter*, and *Salmonella* (79)*.* Infection with enteric bacteria has been associated with diarrheal disease, iron-deficiency anaemia, and growth impairment (80, 81). Poor hygienic behaviors disrupt the fecal-oral route by which many parasitic infections occur (71, 82). A similar pattern of hygiene indicator behavioral variables co-occurrences appeared using association rule learning for underweight and thinness. Furthermore, association rule learning was able to identify sets of co-occurring variables related malnutrition that was otherwise missed using traditional statistical methods. In this study, association rule learning highlighted the importance of different sets of hygiene variables as risk factor of malnutrition by grouping related variables together.

In subgroup analysis by residence, we observed older age, owning a household pet, and vaccination status as important factors for stunting, underweight, and thinness in the urban population, while a house floor made of dust, farm animals, and deworming were important in the suburban/rural population. These differences are corroborated by earlier reports (83–85), that documented rural–urban differentials in factors associated with nutritional outcomes, and linked this divide to socioeconomic inequality and differences in the standards of living of the residents in the two different settings. Previously in Ethiopia, higher odds of undernutrition have been associated with older children in urban locations (58, 86, 87). Older children in urban areas may spend more time outside of their household, increasing their exposure to potential infectious diseases. Owning a housepet may be associated with increased odds of undernutrition through increased exposure to helminth infection. Indeed, Misikir et al., (2020) found a positive association between living with domesticated animals and hookworm infection in a nearby region of Ethiopia (88). Having received vaccinations may be especially important in the urban context where individuals are frequently coming into contact with each other (89). This may be exacerbated by studies that have shown a high degree of vaccine hesitancy among Ethiopians (90, 91), since low levels of vaccination allow for a greater prevalence of infectious diseases.

In the suburban/rural context, house floor material is an indicator of socioeconomic status. Previous studies in Ethiopia have found that socioeconomic status indicators have been associated with increased odds of undernutrition in rural settings (92, 93). In rural Ethiopia, lower socioeconomic status has been associated with a lower dietary diversity score (94), suggesting a potential mechanism by which Ethiopian children of lower socioeconomic status are more likely to be malnourished. Owning farm animals may result in increased odds of undernutrition among children since these animals serve as a reservoir for many infectious agents such as echinococcus (95), cryptosporidium, and giardia (96). The importance of deworming in suburban/rural areas may be a result of increased opportunities for helminth infection. For example, individuals living in these areas are more likely to engage in agriculture, which increases ones susceptibility to hookworm infection (97). Additionally, evidence suggests that Ethiopians living in more rural regions may have poor latrine quality (98), which has been associated with helminth infection since increased exposure to helminths in feces is more likely to result in infection (74). Therefore, long durations without deworming are more likely to have negative consequences in rural contexts where helminth infection and re-infection rates may be higher. The use of subset analysis demonstrates how there is a need to investigate how these larger determinants of health may result in specific predictors of undernutrition. Identifying a unique set of factors contributing to malnutrition across subgroup of population either by residences, age, or sex categories provide insights into planning targeted public health interventions tailored to specific subgroups.

## Limitations

Our findings must be understood considering the following limitations. First, given the cross-sectional design of this study, we are unable to attribute causality between associated exposure and outcome variables. Since we cannot attribute causality, there may be confounding factors for variables that were significantly associated with undernutrition outcomes. Also, the relatively low prevalence of stunting, underweight, and thinness in our study population may have interfered with our ability to detect associations between features and stunting, underweight, and thinness outcomes. Moreover, computation times for subset-based feature selection and association rule learning increase exponentially with the number of variables in a dataset, which limits the applicability of our code to datasets with a large number of risk factors.

Feature selection and association rule learning are two commonly used techniques in machine learning and data analysis; however, both have limitations. Since feature selection reduces the size of the data to enable a more efficient analysis, this method can suffer from overfitting and may not generalize well to new data. Association rule learning can generate a large number of rules, which makes it difficult to extract useful information. Since we focused on association rules of three variables in the interest of computation time and interpretability, it is possible that we overlooked important rules of other sizes. These rules also lacked statistical power since a small proportion of the overall population exhibited undernutrition. This resulted in a relatively small number of individuals from which rules could be generated. However, this lack of power is unlikely to affect our main findings since these association rules supported our feature selection analysis which had high statistical power. Also, association rule learning assumes independence between features, which may not be true in certain instances. Overall, both feature selection and association rule learning are useful tools, but they must be used with caution and in conjunction with other techniques to ensure accurate and robust results.

## Conclusion

In this study, we found that feature selection, association rule learning, and subset analysis substantiated traditional logistic regression findings. As logistic regression identified older age children (10–18 years old) as a risk factor for stunting and underweight, and being female and having received vaccinations as protective factors for stunting, feature selection also found age to be important for stunting and underweight while sex was important for stunting. Feature selection also identified school latrine cleanliness, large family size, and nail trimming habits as novel variables important to stunting, underweight, and thinness outcomes. Association rule learning showed co-occurring hygiene and socioeconomic variables were related to malnutrition, which was otherwise missed using traditional statistical methods. We also demonstrate the need to analyze different population subsections, showing the promise that feature selection may have to uncover a unique set of malnutrition risk factors which could be used to plan targeted public health interventions.

## Data Availability

The datasets presented in this study can be found in online repositories. The names of the repository/repositories and accession number(s) can be found below: Our source code is available on GitHub at https://github.com/CJPIV/SR2022Malnutrition.

## References

[1] Popkin BM Corvalan C Grummer-Strawn LM. Dynamics of the double burden of malnutrition and the changing nutrition reality. Lancet. (2020) 395(10217):65–74. 10.1016/S0140-6736(19)32497-3 31852602 PMC7179702

[2] Doak CM Adair LS Bentley M Monteiro C Popkin BM. The dual burden household and the nutrition transition paradox. Int J Obes (Lond). (2005) 29(1):129–36. 10.1038/sj.ijo.0802824 15505634

[3] Doak CM Adair LS Monteiro C Popkin BM. Overweight and underweight coexist within households in Brazil, China and Russia. J Nutr. (2000) 130(12):2965–71. 10.1093/jn/130.12.2965 11110855

[4] Garrett JL Ruel MT. Stunted child-overweight mother pairs: prevalence and association with economic development and urbanization. Food Nutr Bull. (2005) 26(2):209–21. 10.1177/156482650502600205 16060222

[5] Mbogori T Kimmel K Zhang M Kandiah J Wang Y. Nutrition transition and double burden of malnutrition in Africa: a case study of four selected countries with different social economic development. AIMS Public Health. (2020) 7(3):425–39. 10.3934/publichealth.2020035 32968668 PMC7505783

[6] Black RE Victora CG Walker SP Bhutta ZA Christian P de Onis M Maternal and child undernutrition and overweight in low-income and middle-income countries. Lancet. (2013) 382(9890):427–51. 10.1016/S0140-6736(13)60937-X 23746772

[7] World Health Organization. Nutrition landscape information system (NLIS) country profile indicators: Interpretation guide. Geneva: WHO (2010).

[8] Crosby L Davis A Garde M Jayasinghe D Mason F Pizzini M A life free from hunger: tackling child malnutrition. Fairfield, CT: Save the Children UK (2012). https://resourcecentre.savethechildren.net/document/life-free-hunger-tackling-child-malnutrition/

[9] Larson-Nath C Goday P. Malnutrition in children with chronic disease. Nutr Clin Pract. (2019) 34(3):349–58. 10.1002/ncp.10274 30963628

[10] Rashmi MR Shweta BM Fathima FN Agrawal T Shah M Sequeira R. Prevalence of malnutrition and relationship with scholastic performance among primary and secondary school children in two select private schools in bangalore rural district (India). Indian J Community Med. (2015) 40(2):97–102. 10.4103/0970-0218.153871 25861170 PMC4389510

[11] Martins VJ Toledo Florêncio TM Grillo LP do Carmo PFM Martins PA Clemente AP Long-lasting effects of undernutrition. Int J Environ Res Public Health. (2011) 8(6):1817–46. 10.3390/ijerph8061817 21776204 PMC3137999

[12] Pelletier DL Frongillo EA Jr. Schroeder DG Habicht JP. The effects of malnutrition on child mortality in developing countries. Bull World Health Organ. (1995) 73(4):443–8. 7554015 PMC2486780

[13] Corkins MR. Why is diagnosing pediatric malnutrition important? Nutr Clin Pract. (2017) 32(1):15–8. 10.1177/0884533616678767 27879465

[14] Walton E Allen S. Malnutrition in developing countries. Pediatr Child Health. (2011) 21(9):418–24. 10.1016/j.paed.2011.04.004

[15] UNICEF, WHO, World Bank Group. Levels and trends in child malnutrition. Geneva: World Health Organization (2018). Available at: https://www.who.int/nutgrowthdb/2018-jme-brochure.pdf (Accessed May, 2022).

[16] Chong B Jayabaskaran J Kong G Chan YH Chin YH Goh R Trends and predictions of malnutrition and obesity in 204 countries and territories: an analysis of the global burden of disease study 2019. EClinicalMedicine. (2023) 57:101850. 10.1016/j.eclinm.2023.101850 36864983 PMC9971264

[17] Akombi BJ Agho KE Merom D Renzaho AM Hall JJ. Child malnutrition in sub-saharan Africa: a meta-analysis of demographic and health surveys (2006–2016). PLoS One. (2017) 12(5):e0177338. 10.1371/journal.pone.0177338 28494007 PMC5426674

[18] Ethiopia FDRo. Source: the federal democratic republic of Ethiopia (2016) implementation plan (2016–2030) seqota declaration. Addis Ababa: FDRE (2016).

[19] Abate KH Belachew T. Care and not wealth is a predictor of wasting and stunting of “the coffee kids” of jimma zone, southwest Ethiopia. Nutr Health. (2017) 23(3):193–202. 10.1177/0260106017706253 28641475

[20] Amare D Negesse A Tsegaye B Assefa B Ayenie B. Prevalence of undernutrition and its associated factors among children below five years of age in bure town, west gojjam zone, amhara national regional state, northwest Ethiopia. Adv Public Health. (2016) 2016:7145708. 10.1155/2016/7145708

[21] Edris M. Assessment of nutritional status of preschool children of Gumbrit, North West Ethiopia. Ethiop J Health Dev. (2007) 21:125–9. 10.4314/ejhd.v21i2.10039

[22] Eticha K, editor. Prevalence and determinants of child malnutrition in gimbi district, oromia region, Ethiopia comparative cross—sectional study. AAU Institutional Repository (2007) p. 1–50.

[23] Fentahun W Wubshet M Tariku A. Undernutrition and associated factors among children aged 6–59 months in east belesa district, northwest Ethiopia: a community based cross-sectional study. BMC Public Health. (2016) 16(1):506. 10.1186/s12889-016-3180-0 27297078 PMC4906918

[24] Abate KH Belachew T. Chronic malnutrition among under five children of Ethiopia may not be economic. A systematic review and meta-analysis. Ethiop J Health Sci. (2019) 29(2):265–77. 10.4314/ejhs.v29i2.14 31011275 PMC6460457

[25] Abdulahi A Shab-Bidar S Rezaei S Djafarian K. Nutritional status of under five children in Ethiopia: a systematic review and meta-analysis. Ethiop J Health Sci. (2017) 27(2):175–88. 10.4314/ejhs.v27i2.10 28579713 PMC5440832

[26] Ranganathan P Pramesh CS Aggarwal R. Common pitfalls in statistical analysis: logistic regression. Perspect Clin Res. (2017) 8(3):148–51. 10.4103/picr.PICR_87_17 28828311 PMC5543767

[27] Camacho DM Collins KM Powers RK Costello JC Collins JJ. Next-Generation machine learning for biological networks. Cell. (2018) 173(7):1581–92. 10.1016/j.cell.2018.05.015 29887378

[28] Sharma V Sharma V Khan A Wassmer DJ Schoenholtz MD Hontecillas R Malnutrition, health and the role of machine learning in clinical setting. Front Nutr. (2020) 7:44. 10.3389/fnut.2020.00044 32351968 PMC7174626

[29] Tomar D. A survey on data mining approaches for healthcare. Int J Bio-Sci Bio-Technol. (2013) 5:241–66. 10.14257/ijbsbt.2013.5.5.25

[30] AbdelRahman SE Zhang M Bray BE Kawamoto K. A three-step approach for the derivation and validation of high-performing predictive models using an operational dataset: congestive heart failure readmission case study. BMC Med Inform Decis Mak. (2014) 14:41. 24886637 10.1186/1472-6947-14-41 PMC4074427

[31] Alghamdi M Al-Mallah M Keteyian S Brawner C Ehrman J Sakr S. Predicting diabetes mellitus using SMOTE and ensemble machine learning approach: the henry ford ExercIse testing (FIT) project. PLoS One. (2017) 12(7):e0179805. 10.1371/journal.pone.0179805 28738059 PMC5524285

[32] Aschwanden D Aichele S Ghisletta P Terracciano A Kliegel M Sutin AR Predicting cognitive impairment and dementia: a machine learning approach. J Alzheimers Dis. (2020) 75(3):717–28. 10.3233/JAD-190967 32333585 PMC7934087

[33] Chatterjee A Gerdes MW Martinez SG. Identification of risk factors associated with obesity and overweight-A machine learning overview. Sensors (Basel). (2020) 20(9):2374–54. 10.3390/s20092734 32403349 PMC7248873

[34] Zafar A Attia Z Tesfaye M Walelign S Wordofa M Abera D Machine learning-based risk factor analysis and prevalence prediction of intestinal parasitic infections using epidemiological survey data. PLoS Negl Trop Dis. (2022) 16(6):e0010517. 10.1371/journal.pntd.0010517 35700192 PMC9236253

[35] Bitew FH Sparks CS Nyarko SH. Machine learning algorithms for predicting undernutrition among under-five children in Ethiopia. Public Health Nutr. (2022) 25(2):269–80. 10.1017/S1368980021004262 34620263 PMC8883776

[36] Fenta HM Zewotir T Muluneh EK. A machine learning classifier approach for identifying the determinants of under-five child undernutrition in Ethiopian administrative zones. BMC Med Inform Decis Mak. (2021) 21(1):291. 10.1186/s12911-021-01652-1 34689769 PMC8542294

[37] Khare S. Investigation of nutritional Status of children based on machine learning techniques using Indian demographic and health survey data. Bengaluru: Elsevier (2017).

[38] Mansur M Afiaz A Hossain MS. Sociodemographic risk factors of under-five stunting in Bangladesh: assessing the role of interactions using a machine learning method. PLoS One. (2021) 16(8):e0256729. 10.1371/journal.pone.0256729 34464402 PMC8407547

[39] Rahman SMJ Ahmed N Abedin MM Ahammed B Ali M Rahman MJ Investigate the risk factors of stunting, wasting, and underweight among under-five Bangladeshi children and its prediction based on machine learning approach. PLoS One. (2021) 16(6):e0253172. 10.1371/journal.pone.0253172 34138925 PMC8211236

[40] Kost R Littenberg B Chen ES. Exploring generalized association rule mining for disease co-occurrences. AMIA Annu Symp Proc. (2012) 2012:1284–93. 23304407 PMC3540474

[41] World Health Organization. Growth reference 5–19 years-application tools: anthroplus software. Geneva: World Health Organization (2016).

[42] Boyer SN Wazer DE Band V. E7 protein of human papilloma virus-16 induces degradation of retinoblastoma protein through the ubiquitin-proteasome pathway. Cancer Res. (1996) 56(20):4620–4. 8840974

[43] Wang Y Singh L. Analyzing the impact of missing values and selection bias on fairness. Int J Data Sci Anal. (2021) 12(2):101–19. 10.1007/s41060-021-00259-z

[44] Malarvizhi R Thanamani S. K-Nearest neighbor in missing data imputation. Int J Eng Res Technol. (2012) 5(1):05–7. 10.1007/s10489-015-0666-x

[45] Zeng G. On the analytical properties of category encodings in logistic regression. Commun Stat Theory Methods. (2023) 52(6):1870–87. 10.1080/03610926.2021.1939382

[46] Benjamini Y Hochberg Y. Controlling the false discovery rate: a practical and powerful approach to multiple testing. J R Stat Soc Series B Stat Methodol. (1995) 57(1):289–300. 10.1111/j.2517-6161.1995.tb02031.x

[47] Senan EM Abunadi I Jadhav ME Fati SM. Score and correlation coefficient-based feature selection for predicting heart failure diagnosis by using machine learning algorithms. Comput Math Methods Med. (2021) 2021:8500314. 10.1155/2021/8500314 34966445 PMC8712170

[48] Chen L Li J Zhang YH Feng K Wang S Zhang Y Identification of gene expression signatures across different types of neural stem cells with the Monte-Carlo feature selection method. J Cell Biochem. (2018) 119(4):3394–403. 10.1002/jcb.26507 29130544

[49] Xu Y Huang F Guo W Feng K Zhu L Zeng Z Characterization of chromatin accessibility patterns in different mouse cell types using machine learning methods at single-cell resolution. Front Genet. (2023) 14:1145647. 10.3389/fgene.2023.1145647 36936430 PMC10014730

[50] Zhu JH Yan QL Wang JW Chen Y Ye QH Wang ZJ The key genes for perineural invasion in pancreatic ductal adenocarcinoma identified with monte-carlo feature selection method. Front Genet. (2020) 11:554502. 10.3389/fgene.2020.554502 33193628 PMC7593847

[51] Dramiński M Rada-Iglesias A Enroth S Wadelius C Koronacki J Komorowski J. Monte carlo feature selection for supervised classification. Bioinformatics. (2007) 24(1):110–7. 10.1093/bioinformatics/btm486 18048398

[52] Rathore S Gupta A. A comparative study of feature-ranking and feature-subset selection techniques for improved fault prediction. In: Proceedings of the 7th India Software Engineering Conference, Chennai. Jabalpur (2014).

[53] Kulshreshtha S. Apriori algorithm in R programming. Vellore: GeeksforGeeks (2021).

[54] Soetewey A. Fisher’s exact test in R: independence test for a small sample. Ottignies-Louvain-la-Neuve: Stats and R (2020).

[55] Breiman L. Classification and regression trees. 1st ed. New York, NY: Routledge (1984). 10.1201/9781315139470

[56] Bantie GM Aynie AA Akenew KH Belete MT Tena ET Gebretsadik GG Prevalence of stunting and associated factors among public primary school pupils of Bahir Dar city, Ethiopia: school-based cross-sectional study. PLoS One. (2021) 16(4):e0248108. 10.1371/journal.pone.0248108 33844683 PMC8041191

[57] Getaneh Z Melku M Geta M Melak T Hunegnaw MT. Prevalence and determinants of stunting and wasting among public primary school children in gondar town, northwest, Ethiopia. BMC Pediatr. (2019) 19(1):207. 10.1186/s12887-019-1572-x 31238889 PMC6591879

[58] Herrador Z Sordo L Gadisa E Moreno J Nieto J Benito A Cross-sectional study of malnutrition and associated factors among school aged children in rural and urban settings of Fogera and Libo Kemkem districts, Ethiopia. PLoS One. (2014) 9(9):e105880. 10.1371/journal.pone.0105880 25265481 PMC4179248

[59] Tariku EZ Abebe GA Melketsedik ZA Gutema BT. Prevalence and factors associated with stunting and thinness among school-age children in arba minch health and demographic surveillance site, Southern Ethiopia. PLoS One. (2018) 13(11):e0206659. 10.1371/journal.pone.0206659 30388149 PMC6214544

[60] Akseer N Al-Gashm S Mehta S Mokdad A Bhutta ZA. Global and regional trends in the nutritional status of young people: a critical and neglected age group. Ann N Y Acad Sci. (2017) 1393(1):3–20. 10.1111/nyas.13336 28436100

[61] Garenne M Thurstans S Briend A Dolan C Khara T Myatt M Changing sex differences in undernutrition of African children: findings from demographic and health surveys. J Biosoc Sci. (2021) 54(5):1–11. 10.1017/S0021932021000468 34488914

[62] Gebre A Reddy PS Mulugeta A Sedik Y Kahssay M. Prevalence of malnutrition and associated factors among under-five children in pastoral communities of afar regional state, northeast Ethiopia: a community-based cross-sectional study. J Nutr Metab. (2019) 2019:9187609. 10.1155/2019/9187609 31275645 PMC6589243

[63] Sewnet SS Derseh HA Desyibelew HD Fentahun N. Undernutrition and associated factors among under-five orphan children in Addis Ababa, Ethiopia, 2020: a cross-sectional study. J Nutr Metab. (2021) 2021:6728497. 10.1155/2021/6728497 34760319 PMC8575616

[64] Nandi A Shet A Behrman JR Black MM Bloom DE Laxminarayan R. Anthropometric, cognitive, and schooling benefits of measles vaccination: longitudinal cohort analysis in Ethiopia, India, and Vietnam. Vaccine. (2019) 37(31):4336–43. 10.1016/j.vaccine.2019.06.025 31227354 PMC6620502

[65] Bazie GW Seid M Egata G. Prevalence and predictors of stunting among primary school children in northeast Ethiopia. J Nutr Metab. (2021) 2021:8876851. 10.1155/2021/8876851 34258057 PMC8253620

[66] Ersino G Zello GA Henry CJ Regassa N. Gender and household structure factors associated with maternal and child undernutrition in rural communities in Ethiopia. PLoS One. (2018) 13(10):e0203914. 10.1371/journal.pone.0203914 30286090 PMC6171833

[67] Soboksa NE Gari SR Hailu AB Mengistie Alemu B. Childhood malnutrition and the association with diarrhea, water supply, sanitation, and hygiene practices in kersa and omo nada districts of Jimma Zone, Ethiopia. Environ Health Insights. (2021) 15:1178630221999635. 10.1177/1178630221999635 33746513 PMC7940723

[68] Tusa BS Weldesenbet AB Kebede SA. Spatial distribution and associated factors of underweight in Ethiopia: an analysis of Ethiopian demographic and health survey, 2016. PLoS One. (2020) 15(12):e0242744. 10.1371/journal.pone.0242744 33259562 PMC7707465

[69] Guerrant RL Oriá RB Moore SR Oriá MO Lima AA. Malnutrition as an enteric infectious disease with long-term effects on child development. Nutr Rev. (2008) 66(9):487–505. 10.1111/j.1753-4887.2008.00082.x 18752473 PMC2562291

[70] Gazi MA Alam MA Fahim SM Wahid BZ Khan SS Islam MO Infection with Escherichia Coli pathotypes is associated with biomarkers of gut enteropathy and nutritional Status among malnourished children in Bangladesh. Front Cell Infect Microbiol. (2022) 12:1–9. 10.3389/fcimb.2022.901324 PMC9299418 35873159

[71] Mahmud MA Spigt M Bezabih AM Dinant GJ Velasco RB. Associations between intestinal parasitic infections, anaemia, and diarrhoea among school aged children, and the impact of hand-washing and nail clipping. BMC Res Notes. (2020) 13(1):1. 10.1186/s13104-019-4871-2 31898526 PMC6941294

[72] Rajoo Y Ambu S Lim YA Rajoo K Tey SC Lu CW Neglected intestinal parasites, malnutrition and associated key factors: a population based cross-sectional study among indigenous communities in sarawak, Malaysia. PLoS One. (2017) 12(1):e0170174. 10.1371/journal.pone.0170174 28095446 PMC5240947

[73] Tiruneh T Geshere G Ketema T. Prevalence and determinants of soil-transmitted helminthic infections among school children at goro primary school, South West Shewa, Ethiopia. Int J Pediatr. (2020) 2020:8612054. 10.1155/2020/8612054 32952576 PMC7482002

[74] Zeynudin A Degefa T Tesfaye M Suleman S Yesuf EA Hajikelil Z Prevalence and intensity of soil-transmitted helminth infections and associated risk factors among household heads living in the peri-urban areas of Jimma town, Oromia, Ethiopia: a community-based cross-sectional study. PLoS One. (2022) 17(9):e0274702. 10.1371/journal.pone.0274702 36107925 PMC9477373

[75] Tadege B Mekonnen Z Dana D Tiruneh A Sharew B Dereje E Assessment of the nail contamination with soil-transmitted helminths in schoolchildren in Jimma Town, Ethiopia. PLoS One. (2022) 17(6):e0268792. 10.1371/journal.pone.0268792 35767573 PMC9242460

[76] Crompton DW Nesheim MC. Nutritional impact of intestinal helminthiasis during the human life cycle. Annu Rev Nutr. (2002) 22:35–59. 10.1146/annurev.nutr.22.120501.134539 12055337

[77] Kiani H Haghighi A Salehi R Azargashb E. Distribution and risk factors associated with intestinal parasite infections among children with gastrointestinal disorders. Gastroenterol Hepatol Bed Bench. (2016) 9 (Suppl 1):S80–S7. 28224033 PMC5310805

[78] Yahya RS Awad SI Kizilbash N El-Baz HA Atia G. Enteric parasites can disturb leptin and adiponectin levels in children. Arch Med Sci. (2018) 14(1):101–6. 10.5114/aoms.2016.60707 29379539 PMC5778414

[79] Asfaw Y Ameni G Medhin G Alemayehu G Wieland B. Infectious and parasitic diseases of poultry in Ethiopia: a systematic review and meta-analysis. Poult Sci. (2019) 98(12):6452–62. 10.3382/ps/pez521 31801311 PMC8913983

[80] Haque MA Platts-Mills JA Mduma E Bodhidatta L Bessong P Shakoor S Determinants of Campylobacter infection and association with growth and enteric inflammation in children under 2 years of age in low-resource settings. Sci Rep. (2019) 9(1):17124. 10.1038/s41598-019-53533-3 31748573 PMC6868199

[81] Taye B Enquselassie F Tsegaye A Amberbir A Medhin G Fogarty A Effect of Helicobacter pylori infection on growth trajectories in young Ethiopian children: a longitudinal study. Int J Infect Dis. (2016) 50:57–66. 10.1016/j.ijid.2016.08.005 27531186

[82] Hughes WT. A tribute to toilet paper. Rev Infect Dis. (1988) 10(1):218–22. 10.1093/clinids/10.1.218 3281220

[83] Mussa R. A matching decomposition of the rural–urban difference in malnutrition in Malawi. Health Econ Rev. (2014) 4(1):11. 10.1186/s13561-014-0011-9 25302140 PMC4160003

[84] Sharaf MF Rashad AS. Regional inequalities in child malnutrition in Egypt, Jordan, and Yemen: a Blinder-Oaxaca decomposition analysis. Health Econ Rev. (2016) 6(1):23. 10.1186/s13561-016-0097-3 27271178 PMC4894857

[85] Yaya S Uthman OA Okonofua F Bishwajit G. Decomposing the rural-urban gap in the factors of under-five mortality in sub-saharan Africa? Evidence from 35 countries. BMC Public Health. (2019) 19(1):616. 10.1186/s12889-019-6940-9 31113395 PMC6528236

[86] Alemu TG Muhye AB Ayele AD. Under nutrition and associated factors among adolescent girls attending school in the rural and urban districts of Debark, Northwest Ethiopia: a community-based comparative cross-sectional study. PLoS One. (2021) 16(8):e0254166. 10.1371/journal.pone.0254166 34398878 PMC8366968

[87] Tewabe T Kamal MM Alam K Quazi A Talukder M Hossain SZ. Factors driving underweight, wasting, and stunting among urban school aged children: evidence from Merawi town, Northwest Ethiopia. PLOS Glob Public Health. (2023) 3(1):e0000586. 10.1371/journal.pgph.0000586 36962941 PMC10021509

[88] Misikir SW Wobie M Tariku MK Bante SA. Prevalence of hookworm infection and associated factors among pregnant women attending antenatal care at governmental health centers in DEMBECHA district, North West Ethiopia, 2017. BMC Pregnancy Childbirth. (2020) 20(1):457. 10.1186/s12884-020-03134-0 32787866 PMC7424972

[89] Neiderud CJ. How urbanization affects the epidemiology of emerging infectious diseases. Infect Ecol Epidemiol. (2015) 5:27060. 10.3402/iee.v5.27060 26112265 PMC4481042

[90] Yohannes S Alemayehu A Woldesenbet YM Tadele T Dangiso D Birhanu M COVID-19 vaccine hesitancy among adults in hawassa city administration, sidama region, Ethiopia: a community-based study. Front Public Health. (2023) 11:1–9. 10.3389/fpubh.2023.1122418 PMC10017993 36935692

[91] Muluneh MD Negash K Tsegaye S Abera Y Tadesse D Abebe S COVID-19 Knowledge. Attitudes, and Vaccine Hesitancy in Ethiopia: A Community-Based Cross-Sectional Study. Vaccines (Basel). (2023) 11(4):1774–97. 10.3390/vaccines11040774 37112686 PMC10140841

[92] Bidira K Tamiru D Belachew T. Anthropometric failures and its associated factors among preschool-aged children in a rural community in southwest Ethiopia. PLoS One. (2021) 16(11):e0260368. 10.1371/journal.pone.0260368 34843555 PMC8629177

[93] Mekonnen H. Malnutrition and its correlates among rural primary school children of fogera district, northwest Ethiopia. J Nutr Disord Ther. (2013) 03:1–7. 10.4172/2161-0509.S12-002

[94] Herrador Z Perez-Formigo J Sordo L Gadisa E Moreno J Benito A Low dietary diversity and intake of animal source foods among school aged children in libo kemkem and fogera districts, Ethiopia. PLoS One. (2015) 10(7):e0133435. 10.1371/journal.pone.0133435 26203904 PMC4512702

[95] Pieracci EG Hall AJ Gharpure R Haile A Walelign E Deressa A Prioritizing zoonotic diseases in Ethiopia using a one health approach. One Health. (2016) 2:131–5. 10.1016/j.onehlt.2016.09.001 28220151 PMC5315415

[96] Kifleyohannes T Nødtvedt A Debenham JJ Terefe G Robertson LJ. Cryptosporidium and giardia in livestock in tigray, Northern Ethiopia and associated risk factors for infection: a cross-sectional study. Front Vet Sci. (2022) 8:1–11. 10.3389/fvets.2021.825940 PMC8795829 35097057

[97] Chelkeba L Mekonnen Z Emana D Jimma W Melaku T. Prevalence of soil-transmitted helminths infections among preschool and school-age children in Ethiopia: a systematic review and meta-analysis. Glob Health Res Policy. (2022) 7(1):9. 10.1186/s41256-022-00239-1 35307028 PMC8935818

[98] Novotný J Humňalová H Kolomazníková J. The social and political construction of latrines in rural Ethiopia. J Rural Stud. (2018) 63:157–67. 10.1016/j.jrurstud.2018.08.003

