# Supplementary material for: Feature selection and association rule learning identify risk factors of malnutrition among Ethiopian schoolchildren
Source: Front Epidemiol. 2023 Jul 6;3:1150619. doi: 10.3389/fepid.2023.1150619 (PMC10910994; doi:10.3389/fepid.2023.1150619)
Supplement: Supplementary file 1 [file Table1.docx]

**Supplementary Table 1.** Comparison of K-NN and random forest imputation reveals a high degree of overlapping values (>85%) across all three undernutrition outcomes. K-NN and random forest imputation were performed for missing stunting, underweight, and thinness values, and then the percentage of overlapping values between imputation methods was calculated.

| **Outcome** | **Overlap** |
| --- | --- |
| Stunting | 90.4% |
| Underweight | 90.1% |
| Thinness | 88.4% |

**Supplementary Table 2.** Univariate and multivariate logistic regression results for stunting. Univariate and multivariate logistic regression were performed on demographic, medical, and behavioral variables with stunting as the outcome.

| **Variable** | **Meaning** | **COR** | **P value (uni)** | **CI-95% (uni)** | **AOR** | **P value (adj. P)** | **CI-95% (multi)** |
| --- | --- | --- | --- | --- | --- | --- | --- |
| **DEMOGRAPHIC FACTORS** |  |  |  |  |  |  |  |
| Age |  |  |  |  |  |  |  |
| Continuous | Age increases | 1.095 | 0.002 | 0.033 - 0.15 | 1.128 | 0.0003 (0.013) | 1.06 - 1.20 |
| Sex |  |  |  | 1.034 – 1.162 |  |  |  |
| 0 | Male | 1.000 |  |  | 1.000 |  |  |
| 1 | Female | 0.658 | 0.005 | 0.49- 0.89 | 0.578 | 0.0011 (0.022) | 0.42 - 0.80 |
| Urban |  |  |  |  |  |  |  |
| 0 | No | 1.000 |  |  | 1.000 |  |  |
| 1 | Yes | 1.031 | 0.872 | 0.72 – 1.51 | 1.253 | 0.2963 (0.751) | 0.83 - 1.93 |
| Primary school educated Mother |  |  |  |  |  |  |  |
| 0 | No | 1.000 |  |  | 1.000 |  |  |
| 1 | Yes | 0.922 | 0.589 | 0.68 – 1.23 | 0.950 | 0.7883 (0.879) | 0.66 - 1.38 |
| Secondary school or higher educated Mother |  |  |  |  |  |  |  |
| 0 | No | 1.000 |  |  | 1.000 |  |  |
| 1 | Yes | 1.004 | 0.979 | 0.72 - 0.33 | 1.136 | 0.5562 (0.835) | 0.74 - 1.73 |
| School latrine clean |  |  |  |  |  |  |  |
| 0 | No | 1.000 |  |  | 1.000 |  |  |
| 1 | Yes | 0.000 | 0.979 | NA – Inf. | 0.000 | 0.9784 (0.978) | NA - 2.47E14 |
| Separated Kitchen |  |  |  |  |  |  |  |
| 0 | No | 1.000 |  |  | 1.000 |  |  |
| 1 | Yes | 1.470 | 0.204 | 0.84 – 2.77 | 1.402 | 0.2996 (0.751) | 0.76 - 2.75 |
| Own radio or TV |  |  |  |  |  |  |  |
| 0 | No | 1.000 |  |  | 1.000 |  |  |
| 1 | Yes | 0.910 | 0.702 | 0.57 – 1.51 | 1.018 | 0.9480 (0.972) | 0.61 - 1.75 |
| Own cattle |  |  |  |  |  |  |  |
| 0 | No | 1.000 |  |  | 1.000 |  |  |
| 1 | Yes | 1.403 | 0.209 | 0.81 – 2.34 | 1.452 | 0.2568 (0.751) | 0.75 - 2.74 |
| Own sheep/goat |  |  |  |  |  |  |  |
| 0 | No | 1.000 |  |  | 1.000 |  |  |
| 1 | Yes | 1.096 | 0.744 | 0.62 – 1.86 | 0.870 | 0.6827 (0.848) | 0.44 - 1.67 |
| Own chicken |  |  |  |  |  |  |  |
| 0 | No | 1.000 |  |  | 1.000 |  |  |
| 1 | Yes | 1.031 | 0.287 | 0.74 – 1.42 | 0.918 | 0.6463 (0.835) | 0.63 - 1.32 |
| Household pet |  |  |  |  |  |  |  |
| 0 | No | 1.000 |  |  | 1.000 |  |  |
| 1 | Yes | 1.313 | 0.074 | 0.97 – 1.77 | 1.359 | 0.0606 (0.414) | 0.99 - 1.87 |
| Latrine Location |  |  |  |  |  |  |  |
| 0 | Inside | 1.000 |  |  | 1.000 |  |  |
| 1 | Outside | 2.048 | 0.345 | 0.57 – 13.07 | 1.622 | 0.5388 (0.835) | 0.42 - 10.73 |
| No potable water in house |  |  |  |  |  |  |  |
| 0 | No | 1.000 |  |  | 1.000 |  |  |
| 1 | Yes | 0.757 | 0.073 | 0.56 – 1.03 | 0.826 | 0.2936 (0.751) | 0.58 - 1.18 |
| Family size |  |  |  |  |  |  |  |
| Continuous | Size increases | 1.024 | 0.552 | 0.95 – 1.11 | 1.020 | 0.6393 (0.835) | 0.94 - 1.11 |
| House floor material |  |  |  |  |  |  |  |
|  | Other material(s) | 1.000 |  |  | 1.000 |  |  |
|  | Dust | 1.276 | 0.115 | 0.94 – 1.73 | 1.146 | 0.4622 (0.835) | 0.80 - 1.65 |
| **BIOLOGICAL FACTORS** |  |  |  |  |  |  |  |
| Sickness in past 2 weeks |  |  |  |  |  |  |  |
| 0 | No | 1.000 |  |  | 1.000 |  |  |
| 1 | Yes | 0.917 | 0.598 | 0.66 – 1.26 | 0.973 | 0.8802 (0.925) | 0.67 - 1.39 |
| Ever had vaccination |  |  |  |  |  |  |  |
| 0 | No | 1.000 |  |  | 1.000 |  |  |
| 1 | Yes | 0.656 | 0.008 | 0.48 – 0.90 | 0.445 | 0.0021 (0.028) | 0.26 - 0.74 |
| BCG scar |  |  |  |  |  |  |  |
| 0 | No | 1.000 |  |  | 1.000 |  |  |
| 1 | Yes | 0.865 | 0.334 | 0.64 – 1.16 | 1.490 | 0.1078 (0.631) | 0.93 - 2.45 |
| Has had rash in past year |  |  |  |  |  |  |  |
| 0 | No | 1.000 |  |  | 1.000 |  |  |
| 1 | Yes | 0.810 | 0.467 | 0.44 – 1.39 | 0.907 | 0.7659 (0.879) | 0.46 - 1.69 |
| Asthma confirmed by doctor |  |  |  |  |  |  |  |
| 0 | No | 1.000 |  |  | 1.000 |  |  |
| 1 | Yes | 0.380 | 0.360 | 0.02 – 2.03 | 0.360 | 0.3602 (0.797) | 0.02 - 2.27 |
| Given deworming pill |  |  |  |  |  |  |  |
| 0 | No | 1.000 |  |  | 1.000 |  |  |
| 1 | Yes | 0.951 | 0.743 | 0.70 – 1.28 | 1.027 | 0.8751 (0.925) | 0.74 - 1.43 |
| Given antibiotics in past 3 mo. |  |  |  |  |  |  |  |
| 0 | No | 1.000 |  |  | 1.000 |  |  |
| 1 | Yes | 0.865 | 0.542 | 0.53 – 1.36 | 0.737 | 0.2938 (0.751) | 0.41 - 1.28 |
| Takes any medication |  |  |  |  |  |  |  |
| 0 | No | 1.000 |  |  | 1.000 |  |  |
| 1 | Yes | 1.380 | 0.339 | 0.69 – 2.61 | 2.148 | 0.0550 (0.414) | 0.96 - 4.64 |
| **BEHAVIORAL FACTORS** |  |  |  |  |  |  |  |
| Nail maintainence |  |  |  |  |  |  |  |
| 0 | No | 1.000 |  |  | 1.000 |  |  |
| 1 | Yes | 0.829 | 0.209 | 0.62 – 1.12 | 0.850 | 0.3115 (0.751) | 0.62 - 1.16 |
| Cooking with electricity |  |  |  |  |  |  |  |
| 0 | Some other source | 1.000 |  |  | 1.000 |  |  |
| 1 | With electricity | 0.733 | 0.047 | 0.54 - 0.99 | 0.781 | 0.1934 (0.751) | 0.54 - 1.13 |
| Don’t treat water |  |  |  |  |  |  |  |
| 0 | Treat | 1.000 |  |  | 1.000 |  |  |
| 1 | Drink direct | 1.235 | 0.328 | 0.82 – 1.92 | 1.138 | 0.5845 (0.835) | 0.72 - 1.83 |
| Using river to clean |  |  |  |  |  |  |  |
| 0 | No | 1.000 |  |  | 1.000 |  |  |
| 1 | Yes | 1.232 | 0.162 | 0.92 – 1.65 | 0.921 | 0.6516 (0.835) | 0.64 - 1.31 |
| Defecates in open field |  |  |  |  |  |  |  |
| 0 | Never | 1.000 |  |  | 1.000 |  |  |
| 1 | At least sometimes | 1.023 | 0.907 | 0.70 – 1.48 | 0.877 | 0.5599 (0.835) | 0.56 - 1.36 |
| Don't use school latrine |  |  |  |  |  |  |  |
| 0 | No | 1.000 |  |  | 1.000 |  |  |
| 1 | Yes | 0.831 | 0.328 | 0.57 – 1.20 | 0.727 | 0.1436 (0.736) | 0.47 - 1.11 |
| Don't use toilet paper |  |  |  |  |  |  |  |
| 0 | No | 1.000 |  |  | 1.000 |  |  |
| 1 | Yes | 1.359 | 0.142 | 0.90 – 2.03 | 1.577 | 0.0553 (0.414) | 0.98 - 2.50 |
| Wash hands with water only after defecation |  |  |  |  |  |  |  |
| 0 | No | 1.000 |  |  | 1.000 |  |  |
| 1 | Yes | 0.995 | 0.978 | 0.70 – 1.40 | 0.702 | 0.4678 (0.835) | 0.28 - 1.91 |
| Wash hands with water and soap after defecation |  |  |  |  |  |  |  |
| 0 | No | 1.000 |  |  | 1.000 |  |  |
| 1 | Yes | 0.928 | 0.661 | 0.67 – 1.30 | 0.730 | 0.5024 (0.835) | 0.30 - 1.92 |
| Eats soil |  |  |  |  |  |  |  |
| 0 | Never | 1.000 |  |  | 1.000 |  |  |
| 1 | At least sometimes | 1.168 | 0.667 | 0.60 – 2.51 | 1.429 | 0.3692 (0.797) | 0.68 - 3.27 |
| Never Washes fruits |  |  |  |  |  |  |  |
| 0 | No | 1.000 |  |  | 1.000 |  |  |
| 1 | Yes | 1.107 | 0.805 | 0.46 – 2.39 | 0.728 | 0.4902 (0.835) | 0.28 - 1.72 |
| Eats raw vegetables |  |  |  |  |  |  |  |
| 0 | Never | 1.000 |  |  | 1.000 |  |  |
| 1 | At least sometimes | 0.922 | 0.611 | 0.68 – 1.26 | 0.919 | 0.6419 (0.835) | 0.65 - 1.31 |
| Ever walks barefoot |  |  |  |  |  |  |  |
| 0 | No | 1.000 |  |  | 1.000 |  |  |
| 1 | Yes | 1.293 | 0.086 | 0.96 – 1.73 | 1.251 | 0.1833 (0.751) | 0.90 - 1.74 |
| Wash hands with water before eating |  |  |  |  |  |  |  |
| 0 | No | 1.000 |  |  | 1.000 |  |  |
| 1 | Yes | 1.073 | 0.756 | 0.68 – 1.65 | 0.604 | 0.7479 (0.879) | 0.02 - 18.77 |
| Wash hands with water and soap before eating |  |  |  |  |  |  |  |
| 0 | No | 1.000 |  |  | 1.000 |  |  |
| 1 | Yes | 0.907 | 0.664 | 0.59 – 1.43 | 0.730 | 0.5024 (0.835) | 0.30 - 1.92 |

**Supplementary Table 3.** Univariate and multivariate logistic regression results for underweight. Univariate and multivariate logistic regression were performed on demographic, medical, and behavioral variables with underweight as the outcome.

| **Variable** | **Meaning** | **COR** | **P value (uni)** | **CI-95% (uni)** | **AOR** | **P value (adj. P)** | **CI-95% (multi)** |
| --- | --- | --- | --- | --- | --- | --- | --- |
| **DEMOGRAPHIC FACTORS** |  |  |  |  |  |  |  |
| Age |  |  |  |  |  |  |  |
| Continuous | Age increases | 1.830 | 0.000 | 1.38 – 2.48 | 1.879 | 7.36E-05 (0.003) | 1.40 - 2.60 |
| Sex |  |  |  |  |  |  |  |
| 0 | Male | 1.000 |  |  | 1.000 |  |  |
| 1 | Female | 0.687 | 0.202 | 0.38 – 1.21 | 0.672 | 0.226 (0.999) | 0.35 - 1.27 |
| Urban |  |  |  |  |  |  |  |
| 0 | No | 1.000 |  |  | 1.000 |  |  |
| 1 | Yes | 0.972 | 0.939 | 0.49 – 2.12 | 0.981 | 0.966 (0.999) | 0.42 - 2.44 |
| Primary school educated Mother |  |  |  |  |  |  |  |
| 0 | No | 1.000 |  |  | 1.000 |  |  |
| 1 | Yes | 1.025 | 0.933 | 0.58 – 1.80 | 0.804 | 0.576 (0.999) | 0.38 - 1.75 |
| Secondary school or higher educated Mother |  |  |  |  |  |  |  |
| 0 | No | 1.000 |  |  | 1.000 |  |  |
| 1 | Yes | 0.821 | 0.545 | 0.42 – 1.51 | 0.609 | 0.270 (0.999) | 0.25 - 1.47 |
| School latrine clean |  |  |  |  |  |  |  |
| 0 | No | 1.000 |  |  | 1.000 |  |  |
| 1 | Yes | 10.096 | 0.104 | 0.39 – 257.24 | 16.290 | 0.100 (0.999) | 0.43 - 619.03 |
| Separated Kitchen |  |  |  |  |  |  |  |
| 0 | No | 1.000 |  |  | 1.000 |  |  |
| 1 | Yes | 0.820 | 0.718 | 0.31 – 2.83 | 0.819 | 0.739 (0.999) | 0.28 - 3.05 |
| Own radio or TV |  |  |  |  |  |  |  |
| 0 | No | 1.000 |  |  | 1.000 |  |  |
| 1 | Yes | 0.859 | 0.740 | 0.38 – 2.32 | 0.896 | 0.831 (0.999) | 0.34 - 2.66 |
| Own cattle |  |  |  |  |  |  |  |
| 0 | No | 1.000 |  |  | 1.000 |  |  |
| 1 | Yes | 0.586 | 0.472 | 0.09 – 2.01 | 0.999 | 0.999 (0.999) | 0.13 - 4.75 |
| Own sheep/goat |  |  |  |  |  |  |  |
| 0 | No | 1.000 |  |  | 1.000 |  |  |
| 1 | Yes | 0.278 | 0.212 | 0.02 – 1.34 | 0.462 | 0.510 (0.999) | 0.02 - 3.16 |
| Own chicken |  |  |  |  |  |  |  |
| 0 | No | 1.000 |  |  | 1.000 |  |  |
| 1 | Yes | 0.588 | 0.145 | 0.27 – 1.15 | 0.548 | 0.144 (0.999) | 0.23 - 1.18 |
| Household pet |  |  |  |  |  |  |  |
| 0 | No | 1.000 |  |  | 1.000 |  |  |
| 1 | Yes | 1.378 | 0.271 | 0.77 – 2.44 | 1.619 | 0.138 (0.999) | 0.85 - 3.06 |
| Latrine Location |  |  |  |  |  |  |  |
| 0 | Inside | 1.000 |  |  | 1.000 |  |  |
| 1 | Outside | ######## | 0.985 | -Inf. - NA | ######## | 0.996 (0.999) | 7.39E-76 - NA |
| No potable water in house |  |  |  |  |  |  |  |
| 0 | No | 1.000 |  |  | 1.000 |  |  |
| 1 | Yes | 0.968 | 0.916 | 0.54 – 1.80 | 1.008 | 0.983 (0.999) | 0.50 - 2.09 |
| Family size |  |  |  |  |  |  |  |
| Continuous | Size increases | 0.997 | 0.972 | 0.85 – 1.16 | 1.012 | 0.890 (0.999) | 0.85- 1.20 |
| House floor material |  |  |  |  |  |  |  |
|  | Other material(s) | 1.000 |  |  | 1.000 |  |  |
|  | Dust | 0.817 | 0.519 | 0.43 – 1.48 | 0.664 | 0.274 (0.999) | 0.31 - 1.36 |
| **BIOLOGICAL FACTORS** |  |  |  |  |  |  |  |
| Sickness in past 2 weeks |  |  |  |  |  |  |  |
| 0 | No | 1.000 |  |  | 1.000 |  |  |
| 1 | Yes | 0.797 | 0.468 | 0.42 – 1.45 | 0.760 | 0.465 (0.999) | 0.35 - 1.56 |
| Ever had vaccination |  |  |  |  |  |  |  |
| 0 | No | 1.000 |  |  | 1.000 |  |  |
| 1 | Yes | 1.274 | 0.468 | 0.68 – 2.53 | 1.051 | 0.934 (0.999) | 0.30 - 3.29 |
| BCG scar |  |  |  |  |  |  |  |
| 0 | No | 1.000 |  |  | 1.000 |  |  |
| 1 | Yes | 1.366 | 0.304 | 0.76 – 2.53 | 1.423 | 0.513 (0.999) | 0.53 - 4.51 |
| Has had rash in past year |  |  |  |  |  |  |  |
| 0 | No | 1.000 |  |  | 1.000 |  |  |
| 1 | Yes | 0.672 | 0.460 | 0.20 – 1.73 | 0.749 | 0.630 (0.999) | 0.20 - 2.21 |
| Asthma confirmed by doctor |  |  |  |  |  |  |  |
| 0 | No | 1.000 |  |  | 1.000 |  |  |
| 1 | Yes | 0.000 | 0.987 | NA – Inf. | 0.000 | 0.995 (0.999) | 0 - 1.24E-206 |
| Given deworming pill |  |  |  |  |  |  |  |
| 0 | No | 1.000 |  |  | 1.000 |  |  |
| 1 | Yes | 1.188 | 0.577 | 0.66 – 2.23 | 1.111 | 0.758 (0.999) | 0.57 - 2.21 |
| Given antibiotics in past 3 mo. |  |  |  |  |  |  |  |
| 0 | No | 1.000 |  |  | 1.000 |  |  |
| 1 | Yes | 1.426 | 0.362 | 0.63 – 2.94 | 1.740 | 0.265 (0.999) | 0.63 - 4.50 |
| Takes any medication |  |  |  |  |  |  |  |
| 0 | No | 1.000 |  |  | 1.000 |  |  |
| 1 | Yes | 0.725 | 0.667 | 0.12 – 2.51 | 0.554 | 0.490 (0.999) | 0.08 - 2.47 |
| **BEHAVIORAL FACTORS** |  |  |  |  |  |  |  |
| Nail maintainence |  |  |  |  |  |  |  |
| 0 | No | 1.000 |  |  | 1.000 |  |  |
| 1 | Yes | 1.202 | 0.531 | 0.68 – 2.16 | 0.933 | 0.840 (0.999) | 0.48 - 1.85 |
| Cooking with electricity |  |  |  |  |  |  |  |
| 0 | Some other source | 1.000 |  |  | 1.000 |  |  |
| 1 | With electricity | 0.757 | 0.366 | 0.41 – 1.36 | 0.728 | 0.379 (0.999) | 0.35 (1.46) |
| Don’t treat water |  |  |  |  |  |  |  |
| 0 | Treat | 1.000 |  |  | 1.000 |  |  |
| 1 | Drink direct | 1.303 | 0.558 | 0.58 – 3.49 | 1.290 | 0.608 (0.999) | 0.52 - 3.73 |
| Using river to clean |  |  |  |  |  |  |  |
| 0 | No | 1.000 |  |  | 1.000 |  |  |
| 1 | Yes | 0.964 | 0.901 | 0.54 – 1.69 | 1.140 | 0.713 (0.999) | 0.56 - 2.29 |
| Defecates in open field |  |  |  |  |  |  |  |
| 0 | Never | 1.000 |  |  | 1.000 |  |  |
| 1 | At least sometimes | 0.526 | 0.125 | 0.21- 1.13 | 0.359 | 0.041 (0.833) | 0.12 - 0.90 |
| Don't use school latrine |  |  |  |  |  |  |  |
| 0 | No | 1.000 |  |  | 1.000 |  |  |
| 1 | Yes | 0.610 | 0.271 | 0.23 – 1.36 | 0.581 | 0.278 (0.999) | 0.20 - 1.46 |
| Don't use toilet paper |  |  |  |  |  |  |  |
| 0 | No | 1.000 |  |  | 1.000 |  |  |
| 1 | Yes | 0.813 | 0.702 | 0.48 – 8.17 | 0.943 | 0.923 (0.999) | 0.25 - 2.85 |
| Wash hands with water only after defecation |  |  |  |  |  |  |  |
| 0 | No | 1.000 |  |  | 1.000 |  |  |
| 1 | Yes | 0.951 | 0.880 | 0.48 – 1.79 | ######## | 0.993 (0.999) | 3.91E-44 - NA |
| Wash hands with water and soap after defecation |  |  |  |  |  |  |  |
| 0 | No | 1.000 |  |  | 1.000 |  |  |
| 1 | Yes | 1.216 | 0.558 | 0.65 – 2.44 | ######## | 0.993 (0.9999) | 3.23E-45 - NA |
| Eats soil |  |  |  |  |  |  |  |
| 0 | Never | 1.000 |  |  | 1.000 |  |  |
| 1 | At least sometimes | 0.973 | 0.964 | 0.33 – 4.18 | 0.705 | 0.639 (0.999) | 0.18 - 3.64 |
| Never Washes fruits |  |  |  |  |  |  |  |
| 0 | No | 1.000 |  |  | 1.000 |  |  |
| 1 | Yes | 0.000 | 0.986 | NA - 36.01 | 0.000 | 0.991 (0.999) | 2.66E-219 - 2.14E15 |
| Eats raw vegetables |  |  |  |  |  |  |  |
| 0 | Never | 1.000 |  |  | 1.000 |  |  |
| 1 | At least sometimes | 0.955 | 0.878 | 0.53 – 1.75 | 0.771 | 0.450 (0.999) | 0.39 - 1.53 |
| Ever walks barefoot |  |  |  |  |  |  |  |
| 0 | No | 1.000 |  |  | 1.000 |  |  |
| 1 | Yes | 1.008 | 0.978 | 0.57 – 1.77 | 1.053 | 0.882 (0.999) | 0.53 - 2.10 |
| Wash hands with water before eating |  |  |  |  |  |  |  |
| 0 | No | 1.000 |  |  | 1.000 |  |  |
| 1 | Yes | 0.975 | 0.953 | 0.39 – 2.12 | 0.821 | 0.999 (0.999) | 5.78E-135 - 0 |
| Wash hands with water and soap before eating |  |  |  |  |  |  |  |
| 0 | No | 1.000 |  |  | 1.000 |  |  |
| 1 | Yes | 1.059 | 0.893 | 0.49 – 2.64 | 0.724 | 0.999 (0.999) | 4.46E-24 - 7.02E+25 |

**Supplementary Table 4.** Univariate and multivariate logistic regression results for thinness. Univariate and multivariate logistic regression were performed on demographic, medical, and behavioral variables with thinness as the outcome.

| **Variable** | **Meaning** | **COR** | **P value (uni)** | **CI-95% (uni)** | **AOR** | **P value (adj. P)** | **CI-95% (multi)** |
| --- | --- | --- | --- | --- | --- | --- | --- |
| **DEMOGRAPHIC FACTORS** |  |  |  |  |  |  |  |
| Age |  |  |  |  |  |  |  |
| Continuous | Age increases | 1.066 | 0.331 | 0.93 – 1.21 | 1.054 | 0.472 (0.998) | 0.91 - 1.22 |
| Sex |  |  |  |  |  |  |  |
| 0 | Male | 1.000 |  |  | 1.000 |  |  |
| 1 | Female | 1.088 | 0.802 | 0.57 – 2.12 | 1.006 | 0.987 (0.998) | 0.48 - 2.14 |
| Urban |  |  |  |  |  |  |  |
| 0 | No | 1.000 |  |  | 1.000 |  |  |
| 1 | Yes | 1.058 | 0.895 | 0.48 – 2.66 | 1.039 | 0.939 (0.998) | 0.41 - 2.92 |
| Primary school educated Mother |  |  |  |  |  |  |  |
| 0 | No | 1.000 |  |  | 1.000 |  |  |
| 1 | Yes | 0.628 | 0.193 | 0.30 – 1.23 | 0.922 | 0.859 (0.998) | 0.38 - 2.29 |
| Secondary school or higher educated Mother |  |  |  |  |  |  |  |
| 0 | No | 1.000 |  |  | 1.000 |  |  |
| 1 | Yes | 1.634 | 0.156 | 0.81 – 3.19 | 1.540 | 0.351 (0.998) | 0.63 - 3.91 |
| School latrine clean |  |  |  |  |  |  |  |
| 0 | No | 1.000 |  |  | 1.000 |  |  |
| 1 | Yes | 4.579 | 0.164 | 0.24 – 27.66 | 10.326 | 0.063 (0.8144) | 0.45 - 94.37 |
| Separated Kitchen |  |  |  |  |  |  |  |
| 0 | No | 1.000 |  |  | 1.000 |  |  |
| 1 | Yes | 0.991 | 0.988 | 0.35 – 4.18 | 0.978 | 0.973 (0.998) | 0.31 - 4.44 |
| Own radio or TV |  |  |  |  |  |  |  |
| 0 | No | 1.000 |  |  | 1.000 |  |  |
| 1 | Yes | 0.676 | 0.426 | 0.28 – 2.01 | 0.514 | 0.238 (0.8144) | 0.18 - 1.72 |
| Own cattle |  |  |  |  |  |  |  |
| 0 | No | 1.000 |  |  | 1.000 |  |  |
| 1 | Yes | 0.733 | 0.673 | 0.12 – 2.46 | 0.293 | 0.202 (0.8144) | 0.03 - 1.63 |
| Own sheep/goat |  |  |  |  |  |  |  |
| 0 | No | 1.000 |  |  | 1.000 |  |  |
| 1 | Yes | 5.563 | 0.144 | 0.69 – 5.05 | 5.563 | 0.010 (0.4194) | 1.37 - 19.65 |
| Own chicken |  |  |  |  |  |  |  |
| 0 | No | 1.000 |  |  | 1.000 |  |  |
| 1 | Yes | 0.687 | 0.355 | 0.29 – 1.45 | 0.485 | 0.143 (0.8144) | 0.17 - 1.20 |
| Household pet |  |  |  |  |  |  |  |
| 0 | No | 1.000 |  |  | 1.000 |  |  |
| 1 | Yes | 1.177 | 0.633 | 0.59 – 2.27 | 1.202 | 0.617 (0.998) | 0.57 - 2.46 |
| Latrine Location |  |  |  |  |  |  |  |
| 0 | Inside | 1.000 |  |  | 1.000 |  |  |
| 1 | Outside | ########## | 0.988 | 0 - NA | 16800000.000 | 0.995 (0.998) | 2.98E-47 - Inf |
| No potable water in house |  |  |  |  |  |  |  |
| 0 | No | 1.000 |  |  | 1.000 |  |  |
| 1 | Yes | 0.908 | 0.783 | 0.97 – 6.42 | 0.720 | 0.438 (0.998) | 0.32 - 1.68 |
| Family size |  |  |  |  |  |  |  |
| Continuous | Size increases | 1.142 | 0.105 | 0.97 – 1.34 | 1.141 | 0.157 (0.814) | 0.94 - 1.36 |
| House floor material |  |  |  |  |  |  |  |
|  | Other material(s) | 1.000 |  |  | 1.000 |  |  |
|  | Dust | 0.534 | 0.121 | 0.23 – 1.13 | 0.562 | 0.232 (0.814) | 0.21 - 1.39 |
| **BIOLOGICAL FACTORS** |  |  |  |  |  |  |  |
| Sickness in past 2 weeks |  |  |  |  |  |  |  |
| 0 | No | 1.000 |  |  | 1.000 |  |  |
| 1 | Yes | 0.748 | 0.456 | 0.33 – 1.55 | 0.837 | 0.681 (0.998) | 0.34 - 1.89 |
| Ever had vaccination |  |  |  |  |  |  |  |
| 0 | No | 1.000 |  |  | 1.000 |  |  |
| 1 | Yes | 0.972 | 0.939 | 0.49 – 2.08 | 1.234 | 0.683 (0.998) | 0.45 - 3.40 |
| BCG scar |  |  |  |  |  |  |  |
| 0 | No | 1.000 |  |  | 1.000 |  |  |
| 1 | Yes | 0.703 | 0.294 | 0.36 – 1.36 | 0.681 | 0.413 (0.998) | 0.28 - 1.77 |
| Has had rash in past year |  |  |  |  |  |  |  |
| 0 | No | 1.000 |  |  | 1.000 |  |  |
| 1 | Yes | 1.009 | 0.988 | 0.24 – 2.89 | 1.206 | 0.803 (0.998) | 0.22 - 4.53 |
| Asthma confirmed by doctor |  |  |  |  |  |  |  |
| 0 | No | 1.000 |  |  | 1.000 |  |  |
| 1 | Yes | 3.043 | 0.297 | 0.16 – 16.78 | 4.974 | 0.219 (0.814) | 0.21 - 54.27 |
| Given deworming pill |  |  |  |  |  |  |  |
| 0 | No | 1.000 |  |  | 1.000 |  |  |
| 1 | Yes | 0.964 | 0.916 | 0.50 – 1.93 | 1.072 | 0.853 (0.998) | 0.52 - 2.29 |
| Given antibiotics in past 3 mo. |  |  |  |  |  |  |  |
| 0 | No | 1.000 |  |  | 1.000 |  |  |
| 1 | Yes | 0.643 | 0.470 | 0.15 – 1.82 | 0.690 | 0.581 (0.998) | 0.15 - 2.28 |
| Takes any medication |  |  |  |  |  |  |  |
| 0 | No | 1.000 |  |  | 1.000 |  |  |
| 1 | Yes | 0.000 | 0.987 | 0 – Inf. | 0.000 | 0.991 (0.998) | 1.59E-223 - 2.88E19 |
| **BEHAVIORAL FACTORS** |  |  |  |  |  |  |  |
| Nail maintainence |  |  |  |  |  |  |  |
| 0 | No | 1.000 |  |  | 1.000 |  |  |
| 1 | Yes | 1.653 | 0.151 | 0.84 – 3.39 | 1.793 | 0.130 (0.814) | 0.86 - 3.94 |
| Cooking with electricity |  |  |  |  |  |  |  |
| 0 | Some other source | 1.000 |  |  | 1.000 |  |  |
| 1 | With electricity | 1.864 | 0.064 | 0.96 – 3.63 | 1.959 | 0.101 (0.814) | 0.88 - 4.45 |
| Don’t treat water |  |  |  |  |  |  |  |
| 0 | Treat | 1.000 |  |  | 1.000 |  |  |
| 1 | Drink direct | 0.773 | 0.548 | 0.35 – 1.93 | 0.931 | 0.883 (0.998) | 0.38 - 2.56 |
| Using river to clean |  |  |  |  |  |  |  |
| 0 | No | 1.000 |  |  | 1.000 |  |  |
| 1 | Yes | 1.017 | 0.960 | 0.52 – 1.97 | 1.217 | 0.638 (0.998) | 0.53 - 2.75 |
| Defecates in open field |  |  |  |  |  |  |  |
| 0 | Never | 1.000 |  |  | 1.000 |  |  |
| 1 | At least sometimes | 1.015 | 0.972 | 0.41 – 2.23 | 1.447 | 0.468 (0.998) | 0.50 - 3.79 |
| Don't use school latrine |  |  |  |  |  |  |  |
| 0 | No | 1.000 |  |  | 1.000 |  |  |
| 1 | Yes | 0.728 | 0.483 | 0.27 – 1.65 | 0.704 | 0.500 (0.998) | 0.23 - 1.84 |
| Don't use toilet paper |  |  |  |  |  |  |  |
| 0 | No | 1.000 |  |  | 1.000 |  |  |
| 1 | Yes | 0.799 | 0.677 | 0.23 – 2.05 | 0.697 | 0.539 (0.9983) | 0.19 - 2.01 |
| Wash hands with water only after defecation |  |  |  |  |  |  |  |
| 0 | No | 1.000 |  |  | 1.000 |  |  |
| 1 | Yes | 0.799 | 0.598 | 0.32 – 1.73 | 0.303 | 0.183 (0.814) | 0.06 - 2.31 |
| Wash hands with water and soap after defecation |  |  |  |  |  |  |  |
| 0 | No | 1.000 |  |  | 1.000 |  |  |
| 1 | Yes | 1.050 | 0.901 | 0.51 – 2.39 | 0.354 | 0.211 (0.814) | 0.08 - 2.48 |
| Eats soil |  |  |  |  |  |  |  |
| 0 | Never | 1.000 |  |  | 1.000 |  |  |
| 1 | At least sometimes | 0.887 | 0.872 | 0.26 – 5.58 | 0.623 | 0.571 (0.998) | 0.14 - 4.42 |
| Never Washes fruits |  |  |  |  |  |  |  |
| 0 | No | 1.000 |  |  | 1.000 |  |  |
| 1 | Yes | 0.000 | 0.983 | 0 – inf. | 0.000 | 0.992 (0.998) | 3.39E-244 - 3.49E+23 |
| Eats raw vegetables |  |  |  |  |  |  |  |
| 0 | Never | 1.000 |  |  | 1.000 |  |  |
| 1 | At least sometimes | 1.441 | 0.348 | 0.70 – 3.29 | 1.208 | 0.659 (0.998) | 0.54 - 2.94 |
| Ever walks barefoot |  |  |  |  |  |  |  |
| 0 | No | 1.000 |  |  | 1.000 |  |  |
| 1 | Yes | 0.848 | 0.625 | 0.43 – 1.63 | 0.939 | 0.873 (0.998) | 0.43 - 2.01 |
| Wash hands with water before eating |  |  |  |  |  |  |  |
| 0 | No | 1.000 |  |  | 1.000 |  |  |
| 1 | Yes | 0.199 | 0.113 | 0.01 – 0.93 | 2600309.240 | 0.998 (0.998) | 0 - NA |
| Wash hands with water and soap before eating |  |  |  |  |  |  |  |
| 0 | No | 1.000 |  |  | 1.000 |  |  |
| 1 | Yes | 5.125 | 0.109 | 1.09 – 91.84 | 9985846.640 | 0.998 (0.998) | 0 - NA |

**Supplementary Table 5.** CART method analysis reveals co-occurring hygiene, age, and family size variables associated with an elevated probability of undernutrition. The CART method was used to create decision trees for stunting, underweight, and thinness outcomes, and transcribed into rules to supplement association rule learning analysis.

| **Stunting** | **Probability** |
| --- | --- |
| ≥10 years old, don’t use school latrine, Family size ≥4 | 56% |
| **Underweight** |  |
| ≥10 years old, family size ≥5, defecate in field | 57% |
| **Thinness** |  |
| Family size ≥8, mother did not complete secondary school, ≥12 years old | 57% |

**Supplementary Table 6.** Association rule learning for population subsets reinforces subset feature selection findings, identifying co-occurring features that significantly increase one’s odds of undernutrition. Association rules were obtained for residency and age population subsets and the top three rules were selected for each outcome. (*Underweight is only calculated for children under 10 years of age).

| **Urban** | support | confidence | lift | OR | P-value |
| --- | --- | --- | --- | --- | --- |
| Stunting |  |  |  |  |  |
| don’t treat water,don’t use toilet paper,wash hands with water after latrine | 0.011 | 0.50 | 2.21 | 3.54 | 0.009 |
| own household pet,house floor made of dust, nail maintenance | 0.018 | 0.47 | 2.07 | 3.19 | 0.002 |
| don't treat water,don't use toilet paper,house floor made of dust | 0.011 | 0.43 | 1.90 | 2.65 | 0.029 |
| Underweight |  |  |  |  |  |
| own household pet,take antibiotics, clean self or clothes in river | 0.006 | 0.25 | 2.76 | 3.52 | 0.086 |
| own household pet,don't treat water,take antibiotics | 0.008 | 0.21 | 2.32 | 2.84 | 0.083 |
| female,own household pet,wash hands with water after latrine | 0.008 | 0.20 | 2.20 | 2.66 | 0.097 |
| Thinness |  |  |  |  |  |
| own household pet,defecate in field,take antibiotics | 0.002 | 0.25 | 6.74 | 9.18 | 0.032 |
| had rash in past two weeks,house floor made of dust,cleans self or clothes in river | 0.002 | 0.20 | 5.39 | 6.87 | 0.050 |
| defecate in field,walk barefoot,take antibiotics | 0.002 | 0.20 | 5.39 | 6.87 | 0.050 |
| **Suburban/rural** |  |  |  |  |  |
| Stunting |  |  |  |  |  |
| own cattle,walk barefoot,sickness in the last two weeks | 0.020 | 0.80 | 3.65 | 15.59 | 0.009 |
| house floor made of dust,take any meds | 0.015 | 0.75 | 3.42 | 11.40 | 0.034 |
| have BCG Scar,house floor made of dust,take any meds | 0.015 | 0.75 | 3.42 | 11.40 | 0.034 |
| Underweight |  |  |  |  |  |
| take antibiotics,house floor made of dust,engage in nail maintenance | 0.019 | 0.67 | 7.00 | 23.50 | 0.023 |
| take antibiotics,house floor made of dust,clean self or clothes in river | 0.019 | 0.67 | 7.00 | 23.50 | 0.023 |
| house has potable water,take antibiotics,clean self or clothes in river | 0.029 | 0.33 | 3.50 | 6.36 | 0.039 |
| Thinness |  |  |  |  |  |
| own sheep or goat,house has potable water,engage in nail maintenance | 0.005 | 0.50 | 16.33 | 37.80 | 0.060 |
| own sheep or goat,house has potable water,walk barefoot | 0.010 | 0.40 | 13.07 | 31.17 | 0.008 |
| have received vaccinations,own sheep or goat,House has potable water | 0.010 | 0.40 | 13.07 | 31.17 | 0.008 |
| **Under 10** |  |  |  |  |  |
| Stunting |  |  |  |  |  |
| don't use toilet paper,house floor made of dust | 0.010 | 0.60 | 2.94 | 6.12 | 0.007 |
| don't use toilet paper,eat soil,house floor made of dust | 0.010 | 0.60 | 2.94 | 6.12 | 0.007 |
| don't use toilet paper,frequency of raw vegetable washing,house floor made of dust | 0.010 | 0.60 | 2.94 | 6.12 | 0.007 |
| Underweight* |  |  |  |  |  |
| defecate in field,walk barefoot,take antibiotics | 0.005 | 0.33 | 3.64 | 5.20 | 0.041 |
| defecate in field,take antibiotics,clean self or clothes in river | 0.005 | 0.27 | 2.98 | 3.89 | 0.071 |
| defecate in field,frequency of raw vegetable washing,take antibiotics | 0.005 | 0.25 | 2.73 | 3.45 | 0.088 |
| Thinness |  |  |  |  |  |
| own household pet,defecate in field,take antibiotics | 0.003 | 0.50 | 16.08 | 34.94 | 0.005 |
| don't use school latrine,wash hands with water after latrine,engage in nail maintenance | 0.003 | 0.33 | 10.72 | 17.41 | 0.013 |
| own household pet,don't use school latrine,wash hands with water after latrine | 0.003 | 0.22 | 7.15 | 9.89 | 0.029 |
| **10 and older** |  |  |  |  |  |
| Stunting |  |  |  |  |  |
| don't treat water,had rash in last two weeks,had sickness in last two weeks | 0.013 | 0.60 | 2.38 | 4.65 | 0.019 |
| don't treat water,don't use toilet paper,wash hands with water after latrine | 0.011 | 0.56 | 2.21 | 3.84 | 0.048 |
| had rash in last two weeks,had sickness in last two weeks,clean self or clothes in river | 0.011 | 0.56 | 2.21 | 3.84 | 0.048 |
| Thinness |  |  |  |  |  |
| own cattle,own sheep or goat,own household pet | 0.004 | 0.25 | 5.96 | 8.39 | 0.040 |
| own cattle,own sheep or goat,eat raw vegetables | 0.004 | 0.20 | 4.77 | 6.26 | 0.061 |
| live in urban area,defecate in field,wash hands with water after latrine | 0.007 | 0.19 | 4.47 | 6.07 | 0.025 |
